# Characterization of recharge components for delineation of a high-alpine rock glacier spring catchment, Ötztal Alps (northern Italy)

**DOI:** 10.1007/s10040-025-03005-y

**Published:** 2026-01-19

**Authors:** Giulia Bertolotti, Matevž Vremec, Simon Seelig, Karl Krainer, Thomas Wagner, Andrea Fischer, Gerfried Winkler

**Affiliations:** 1 Institute for Interdisciplinary Mountain Research, Austrian Academy of Sciences, Innrain 25/3, 6020 Innsbruck, Austria; 2 Department of Earth Sciences, NAWI Graz Geocenter, University of Graz, Heinrichstr. 26, 8010 Graz, Austria; 3 Institute of Geology, University of Innsbruck, Innrain 52, 6020 Innsbruck, Austria; 4 Alma Mater Europaea University, Slovenska 17, Maribor, Slovenia

**Keywords:** Rock glacier, Italy, Rainfall-runoff modeling, Catchment delineation, Mountain hydrology permafrost hydrology

## Abstract

Rock glaciers provide shallow groundwater reservoirs that affect the hydrology of alpine headwater catchments. However, quantifying and identifying their temporally dominating recharge components, as well as delineating their hydrological catchment, can be challenging. The present study makes use of a lumped-parameter rainfall-runoff model (GR4J-CemaNeige) to tackle these issues, applied at a test site in the Ötztal Alps (northern Italy). The model relies on gridded precipitation and air-temperature data and is calibrated using discharge data. Three different catchment scenarios varying in size and glacier coverage extent were modeled to determine the most likely catchment. The model's outcome was validated using oxygen and hydrogen stable isotopes and electrical conductivity analyses. Three dominant recharge components were quantified by the rainfall-runoff model for the 2012–2021 period: snowmelt (607 mm/year, 56%), rainfall (443 mm/year, 42%), and glacial ice melt (25 mm/year, 2%). The rock glacier permafrost ice melt contributes less than 1% of the total annual discharge, falling within the model uncertainty range. The study demonstrates that a simple rainfall-runoff model helps to identify individual recharge components. It gives a crucial indication for the most likely delineation of a rock glacier spring catchment in a complex periglacial and glacial environment. This provides the basis for quantitatively predicting climate change impact on such highly sensitive environments.

**Supplementary Information:**

The online version contains supplementary material available at 10.1007/s10040-025-03005-y.

## Résumé

Les glaciers rocheux fournissent des réservoirs d’eaux souterraines peu profondes qui impactent l’hydrologie des têtes de bassins versants alpins. Cependant, la quantification et l’identification de leurs composantes de recharge qui dominent dans le temps, ainsi que la délimitation de leur bassins versants hydrologiques, peuvent s’avérer difficile. La présente étude fait usage d’un modèle à réservoirs pluie-débit (GR4J-CemaNeige) pour résoudre ces problèmes, appliqué à un site pilote dans les Alpes d’Ötztal (Nord de l’Italie). Le modèle repose sur des données maillées de précipitation et de la température de l’aire et est calibré en utilisant les données de débits. Trois scénarios différents de bassins d’alimentation variant en dimension et en extension de couverture de glacier ont été modélisés pour déterminer le bassin versant le plus probable. Les résultats du modèle ont été validés en utilisant les isotopes stables de l’oxygène et de l’hydrogène et les analyses de la conductivité électrique. Les trois composantes dominantes de la recharge ont été quantifiées par modélisation pluie-débit pour la période 2012-2021 : fonte de neige (607 mm/an, 56%), précipitation (443 mm/an, 42%) et fonte de glace glaciaire (25 mm/an, 2%). La fonte de la glace du permafrost du glacier rocheux contribue à moins de 1% du débit total annuel, ce qui se situe dans la marge d’incertitude du modèle. L’étude démontre qu’un simple modèle pluie-débit aide à identifier les composantes individuelles de la recharge. Il fournit des indications cruciales pour la délimitation la plus probable d’un bassin versant de source d’un glacier rocheux dans un environnement périglaciaire et glaciaire complexe. Cela fournit les bases pour prédire quantitativement l’impact du changement climatique sur ces environnements hautement sensibles.

## Resumen

Los glaciares de roca proporcionan depósitos de agua subterránea poco profundos que afectan a la hidrología de las cuencas hidrográficas alpinas. Sin embargo, cuantificar e identificar sus componentes de recarga temporalmente dominantes, así como delimitar su cuenca hidrográfica, puede resultar problemático. El presente estudio utiliza un modelo de precipitación-escorrentía de parámetros agrupados (GR4J-CemaNeige) para abordar estas cuestiones, aplicado en un lugar de ensayo en los Alpes de Ötztal (norte de Italia). El modelo se basa en datos de precipitación y temperatura del aire en forma de cuadrícula y se calibra utilizando datos de descarga. Se modelaron tres escenarios de cuencas diferentes que variaban en tamaño y extensión de la cobertura glaciar para determinar la cuenca más probable. El resultado del modelo se validó utilizando isótopos estables de oxígeno e hidrógeno y análisis de conductividad eléctrica. El modelo de precipitación-escorrentía cuantificó tres componentes de recarga dominantes para el período 2012-2021: deshielo (607 mm/año, 56 %), precipitaciones (443 mm/año, 42 %) y deshielo glaciar (25 mm/año, 2 %). El deshielo del permafrost de los glaciares de roca contribuye con menos del 1 % del caudal anual total, lo que se encuentra dentro del rango de incertidumbre del modelo. El estudio demuestra que un modelo simple de precipitación-escorrentía ayuda a identificar los componentes individuales de recarga. Proporciona una indicación fundamental para la delimitación más probable de una cuenca hidrográfica de manantiales de glaciares de roca en un entorno periglacial y glacial complejo. Esto proporciona la base para predecir cuantitativamente el impacto del cambio climático en entornos tan sensibles.

## Abstract (zh)

岩石冰川提供影响高山源头集水区水文过程的浅层地下水储库。然而，量化并识别其随时间变化的主导补给分量，以及划定其水文集水区，可能具有挑战性。本研究利用一个集总参数降雨-径流模型（GR4J-CemaNeige）来应对这些问题，并在意大利北部Ötztal阿尔卑斯山的一处试验地点进行应用。该模型依赖网格化降水量与气温数据，并以径流数据进行标定。以三种在规模和冰川覆盖程度上不同的集水区情景进行了建模，以确定最可能的集水区边界。模型结果通过氧同位素与氢同位素稳定同位素分析以及电导率分析进行验证。在2012–2021年期间，降雨-径流模型定量识别出三大主导补给分量：雪融水607 mm/yr，占56%；降雨量443 mm/yr，占42%；冰川融水25 mm/yr，占2%。岩石冰川永久冻土融水对年总径流的贡献不足1%，落在模型不确定性范围内。研究表明，简单的降雨-径流模型有助于识别各自的补给分量。它为在复杂的近冰川冻融与冰川环境中最可能划定岩石冰川泉源集水区提供关键指示，为定量预测气候变化对这类高度敏感环境的影响提供基础。

## Resumo

Geleiras rochosas fornecem reservatórios rasos de águas subterrâneas que afetam a hidrologia das captações das águas de nascente das montanhas. No entanto, quantificar e identificar seus componentes de recarga dominante temporariamente, assim como a delimitação da sua captação hidrológica, podem ser desafiadores. O presente estudo faz o uso de um modelo precipitação-vazão de parâmetro agrupado (GR4J-CemaNeige) para enfrentar tais questões, aplicado à um teste in loco nos Ötztal Alps (Norte da Itália). O modelo se baseia em dados de precipitação e temperatura do ar matriciados e é calibrado utilizando dados de descarga. Três cenários diferentes de captação variando em tamanho e extensão da cobertura da geleira foram determinados para a melhor captação possível. O resultado do modelo foi validado utilizando isótopos estáveis de oxigênio e hidrogênio e análises de condutividade elétrica. Três componentes de recarga dominantes foram quantificados pelo modelo precipitação-vazão no período de 2012-2021: derretimento de neve (607 mm/ano, 56%), precipitação (443 mm/ano, 42%), derretimento de gelo glacial (25mm/ano, 2%). O derretimento do gelo do pergelissolo da rocha glacial contribui para menos de 1% da descarga anual total, diminuindo o alcance da incerteza do modelo. Esse estudo demonstra que um modelo simples de precipitação-vazão ajuda a identificar componentes de recarga individuais. Isso dá uma indicação crucial para uma melhor delimitação da captação de nascente de rocha glacial em um ambiente complexo periglacial e glacial. O estudo fornece a base para previsão quantitativa dos impactos das mudanças climáticas em ambiente extremamente sensíveis.

## Introduction

Climate change is significantly impacting alpine freshwater resources in both quantity and quality. This is primarily due to retreating glaciers, as highlighted by the Intergovernmental Panel on Climate Change (IPCC 2019). At the same time, there is an increasing influence from rock glaciers, paraglacial and periglacial landforms, as indicated by research conducted by e.g., Haeberli et al. (2017), Jones et al. (2019), Brighenti et al. (2019, 2021, 2023), Pruessner et al. (2022) and Wagner et al. (2021a). Runoff changes also impact aquatic downstream ecology in high alpine regions (Brown et al. 2018; Brighenti et al. 2019). Lösch et al. (2015), Nickus et al. (2015) and Thaler et al. (2015) showed for example that creeks and high alpine lakes derived from intact rock glaciers and characterized by low water temperatures, high electrical conductivity (EC), high sulfate, magnesium (Mg) and calcium (Ca), and partly high metal concentrations (close to or above the drinking water limit) display a significantly lower biodiversity compared to high alpine creeks and lakes that are not influenced by water derived from rock glaciers or more generally from permafrost-affected landforms.

Research on the hydrogeology of rock glaciers has intensified in recent decades, with contributions from scholars like Harrington et al. (2018), Hayashi (2020), Krainer et al. (2007), Krainer and Mostler (2002), Winkler et al. (2016), and Wagner et al. (2021b). In the Austrian Alps, existing data reveal a significant volume of water, primarily in the form of ice, stored in rock glaciers and glaciers (Wagner et al. 2021a). Although the unfrozen base layer of rock glaciers currently represents only a small fraction of the total ice water equivalent of glaciers and rock glaciers, their storage capacity is expected to increase as englacial ice within the rock glacier thaws, creating more pore space available for groundwater storage (e.g., Rogger et al. 2017; Wagner et al. 2021b). Water stored and released from intact rock glaciers originates from various sources, including snowmelt, rainfall, (rock glacier) permafrost ice melt, and meltwater from cirque glaciers within the catchment (Krainer and Mostler 2002; Wagner et al. 2021b; Bertolotti 2022). This is reflected by both seasonal and daily variations in discharge. Seasonal discharge variations mirror the availability of water, with cold water temperatures (due to ice contact and melting snow) and high discharge during the snowmelt period and storm events in the summer months, and lower discharge with increased electrical conductivity in winter and autumn (Krainer et al. 2015a; Tolotti et al. 2013; Giardino et al. 2011). Standard hydrogeological tools have proven effective for the identification and quantification of various sources of recharge as well as different discharge components from rock glaciers (e.g., Wagner et al. 2021b). These tools encompass a range of methods, including geochemical analyses, the application of conservative tracers such as stable isotopes (δ1⁸O, δ2H) and major ions, and hydrological modeling approaches. Stable isotope analysis has been widely used to distinguish between snowmelt, glacier ice melt, and groundwater contributions in glaciated alpine environments (e.g., Giustini et al. 2016; Longinelli and Selmo 2003; Winkler et al. 2016; Brighenti et al. 2023; Delpero et al. 2025). Lumped parameter models—such as mixing models and reservoir models—are frequently employed to interpret discharge dynamics and quantify the relative contributions of distinct water sources over time (e.g., Perrin et al. 2003; Wagner et al. 2016, 2021b). Hydrological modeling tools, such as the GR4J model combined with snow and ice melt modules (e.g., GR4J-CemaNeige), have proven useful for simulating catchment runoff, distinguishing between recharge components, and delineating hydrologically active areas (Valéry et al. 2014; Wagner et al. 2021b). However, hydrological models rely on meteorological data that are scarce in mountain regions, leading to the use of gridded datasets that may miss local variability and introduce uncertainties in discharge modeling (Hiebl and Frei 2016; Vremec et al. 2025). These integrated approaches are particularly valuable for improving the process-based understanding of rock glacier hydrology and assessing their evolving role in alpine water budgets under changing climatic conditions (Hayashi 2020; Arenson et al. 2022; Brighenti et al. 2024).

In high-alpine regions, the delineation of spring catchments is still challenging, especially if these are affected by glacial and periglacial landforms that may hide the watershed outline (Arenson et al. 2022). In particular, this is true for rock glacier springs with high discharge from large and hydrogeologically complex catchments. The active rock glacier Lazaun (Schnals Valley/Val Senales, Italy) was selected for this study because of investigations since 2006, particularly concerning composition, internal structure, ice content, and flow velocity (e.g., Bressan 2007; Krainer et al. 2015a, b; Fey and Krainer 2020; Bertolotti 2022; Bertone et al. 2023). More recently, investigations concerning the ecology and hydrochemistry (Brighenti et al. 2023; Delpero et al. 2025) and hydrogeology (Bertolotti 2022; Brighenti et al. 2023) of its catchment and the nearby area have intensified. None of the previous studies, however, concentrated on the delineation of the rock glacier catchment per se and on the characterization of its discharge components. This is important information for a better understanding of rock glacier hydrology, considering the complex glacial and periglacial environment, and for assessing future hydrodynamics of high alpine catchments in general.

Thus, the aims of this study are (i) to quantify the predominant recharge components, including rain, snowmelt, and potentially glacier ice melt, and (ii) to delineate the catchment area of a rock glacier spring in a hydro(geo)logically complex glacial and periglacial environment. This was primarily achieved through the adaptation and modification of a lumped-parameter rainfall-runoff model (GR4J; Perrin et al. 2003), with added modules to account for snow and ice melt.

## Study area

### Geomorphology and geology

**Fig. 1:**
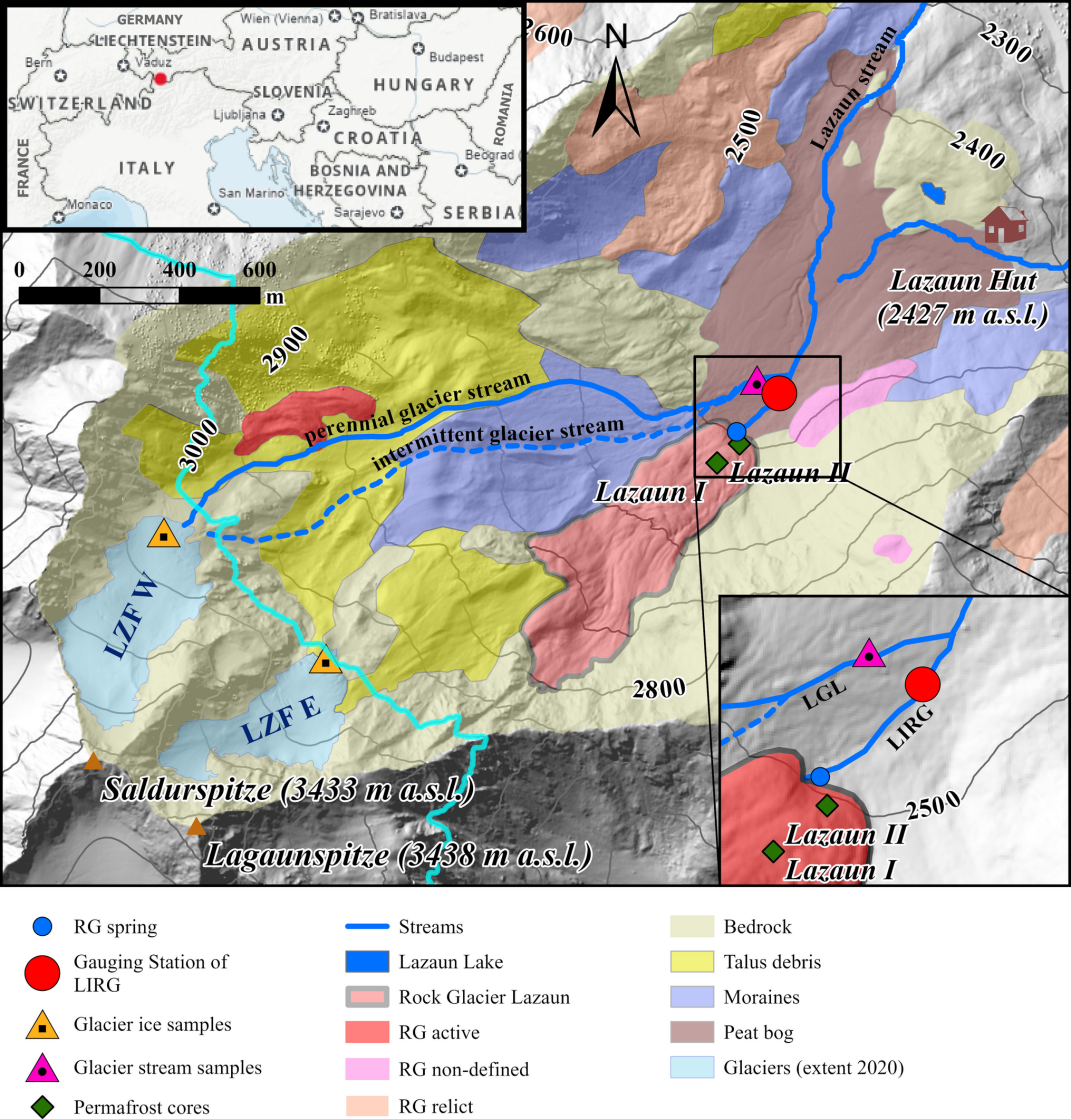
Study area. The* upper left* insert shows the study area (*red dot*) in relation to South Tyrol (*orange polygon*). The main map shows an overview of the Lazaun cirque with the location of the active Rock Glacier Lazaun, its spring and the related gauging station. Other rock glaciers and their cryospheric status are also depicted, according to the rock glacier inventory of the Autonomous Province of Bolzano (GeoKatalog 2021). Two cirque glaciers (Lazaunferner West (LZF W) and Lazaunferner East (LZF E)) are also highlighted, as well as the location for ice sampling, sampling from the glacier stream (LGL) and the locations of the permafrost cores by Krainer et al. 2015a (cf. Monitoring and sampling). The extension of bedrock, moraine material, talus debris, and the peat bog area are also presented, as mapped by Bertolotti (2022). DEM version 2006 from GeoKatalog (2021). The* lower right* insert highlights the confluence of LGL and the rock glacier stream (LIRG) into the Lazaun stream downstream of the gauging station. LGL is divided into two arms upstream of its confluence into LIRG: a “perennial” and an “intermittent” glacier stream. The intermittent glacier stream runs dry in periods of low snow and ice melt (cf. Section 2)

The study site is located at Lazaun cirque, a north-northeast facing cirque in the upper Schnals Valley/Val Senales west of Kurzras/Maso Corto in the southern Ötztal Alps (Autonomous Province Bozen/Bolzano – Südtirol/South Tyrol, northern Italy at 46°44´49´´N and 10°45´20´´E, Fig. 1). Spanning from 2450 to 3400 m in elevation, the Lazaun cirque lies entirely above the treeline and falls within the domain characterized by permafrost (Gärtner-Roer et al. 2010), as indicated by the Alpine Permafrost Index Map (APIM; Boeckli et al. 2012). However, the term “permafrost” and “permafrost ice” will refer hereafter only to the permafrost ice within Rock Glacier Lazaun (also “englacial ice”). The quantification of permafrost ice or ground ice within the rest of the catchment (in talus debris and moraines; Fig. 1) is more complex and uncertain and thus beyond the scope of this paper.

The bedrock of the topographic catchment area is mainly composed of polymetamorphic paragneiss (biotite-plagioclase gneiss) and mica schist (Krainer et al. 2015b; Bertolotti 2022), rarely of orthogneiss of the Ötztal-Stubai Metamorphic Complex of the Austroalpine Ötztal-Bundschuh Nappe System (Hoinkes and Thöni 1993; Hoinkes et al. 2021). The crystalline bedrock within the Lazaun cirque is clearly visible in several outcrops and sums up to nearly half of the cirque. Otherwise, the cirque is covered by talus, moraine material, and rock glaciers (Fig. 1). Information about thickness and composition of the sediments in the cirque outside of the Rock Glacier Lazaun is not available and thus little information can be derived about the subsurface geometry of the cirque.

The cirque hosts two intact rock glaciers, two relict rock glaciers and one or two rock glaciers of non-defined activity, according to the 2010 rock glacier inventory of the Geographic Information Service of the Autonomous Province of Bolzano (GeoKatalog 2021). The largest rock glacier is the active Rock Glacier Lazaun (Fig. 1). It is a medium-sized, tongue-shaped rock glacier that is 814 m long, up to 320 m wide and covers an area of 0.17 km^2^. The rock glacier extends from an elevation of approximately 2800 m at the rooting zone to 2480 m at the base of the steep front (Krainer et al. 2015a, b; GeoKatalog 2021). The surface layer (i.e., “active layer”) of the rock glacier is generally coarse-grained. At finer-grained areas, clasts with diameters up to 10 cm dominate. At coarse-grained sites, clasts up to 30 cm in diameter are most abundant, larger clasts are subordinate (Krainer et al. 2015a).

In 2010, two cores were drilled on the lower part of the rock glacier down to depths of 40 and 32 m (Fig. 1), respectively providing information on the internal structure, ice content, and isotopic signature of permafrost ice (Krainer et al. 2015a, b). According to Krainer et al. (2015b), the frozen core of the rock glacier covers an area of approximately 0.1 km^2^ and the annual melting rate of the rock glacier ice (derived from GPS measurements) is in the order of 10 cm on average resulting in a total ice volume of 10,000 m^3^ (approximately 9100 m^3^ of water equivalent) lost from the rock glacier by melting each year during May until October (6 months).

The Lazaun cirque also hosts two small cirque glaciers upstream of Rock Glacier Lazaun, the Lazaunferner West (LZF W) and the Lazaunferner East (LZF E) (Fig. 1). The glaciers are similar in size (LZF W (2020): 0.11 km^2^, LZF E (2020): 0.07 km^2^; cfr). See Table S1 of the electronic supplementary material (ESM) for additional details, but smaller than the Rock Glacier Lazaun (0.17 km^2^). A peat bog stretches between the Rock Glacier Lazaun and the Lazaun hut (located at the eastern end of the cirque, downstream of the rock glacier; Fig. 1).

### Hydrology

Starting downstream, the peat bog is traversed by the Lazaun stream. The Lazaun stream is fed both by the stream originating at Rock Glacier Lazaun (LIRG, “Lazaun Intact Rock Glacier stream”) and by a glacier stream (LGL, “Lazaun Glacier stream”) (Fig. 1). LGL is divided in two arms which merge just before their common confluence into the Lazaun stream (inset in Fig. 1). The northernmost arm (“perennial glacier stream” in Fig. 1) can be entirely followed superficially up to Lazaunferner West, whereas the southernmost arm (“intermittent glacier stream” in Fig. 1) disappears partially under the talus and moraine debris and runs dry in periods of low snow and glacial melt (e.g., in late summer). No stream originating at Lazaunferner East could be identified. The intermittent flow of the southernmost arm of the LZF W glacier stream suggests that some subsurface flow also towards the Rock Glacier Lazaun is possible. This has to be considered when discussing the water balance of the rock glacier spring.

**Fig. 2:**
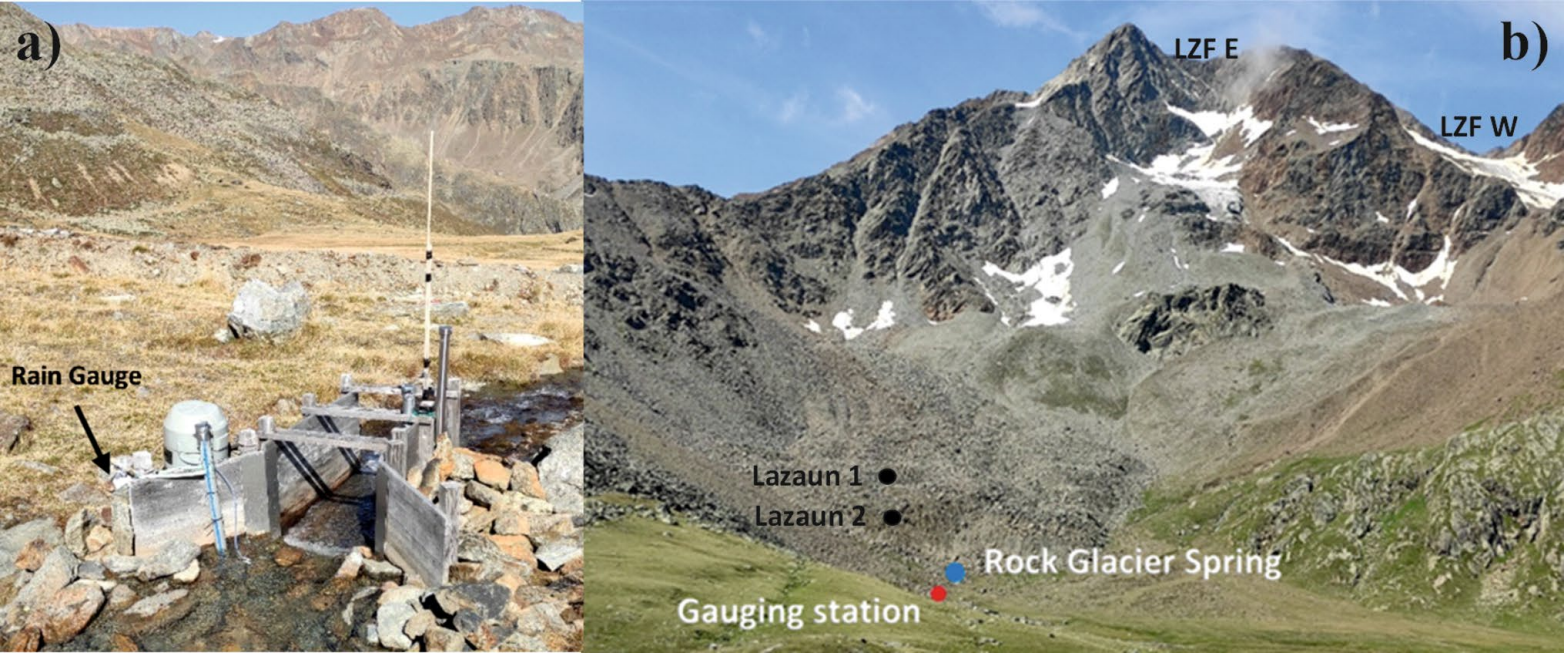
Field impressions: (**A**) The gauging station, with the datalogger (*blue-grey*) measuring water level, temperature, and EC, the automatic water sampler for daily isotope samples (cf. Monitoring and sampling) and a rain gauge. The gauging station is located approximately 100 m downstream from the rock glacier spring, as shown in the panorama picture of the cirque (**B**), with Rock Glacier Lazaun in the front and the two cirque glaciers in the background; see also Fig. 1. The position of the two rock glacier cores (Lazaun 1 and 2) is also shown in (**B**)

**Fig. 3:**
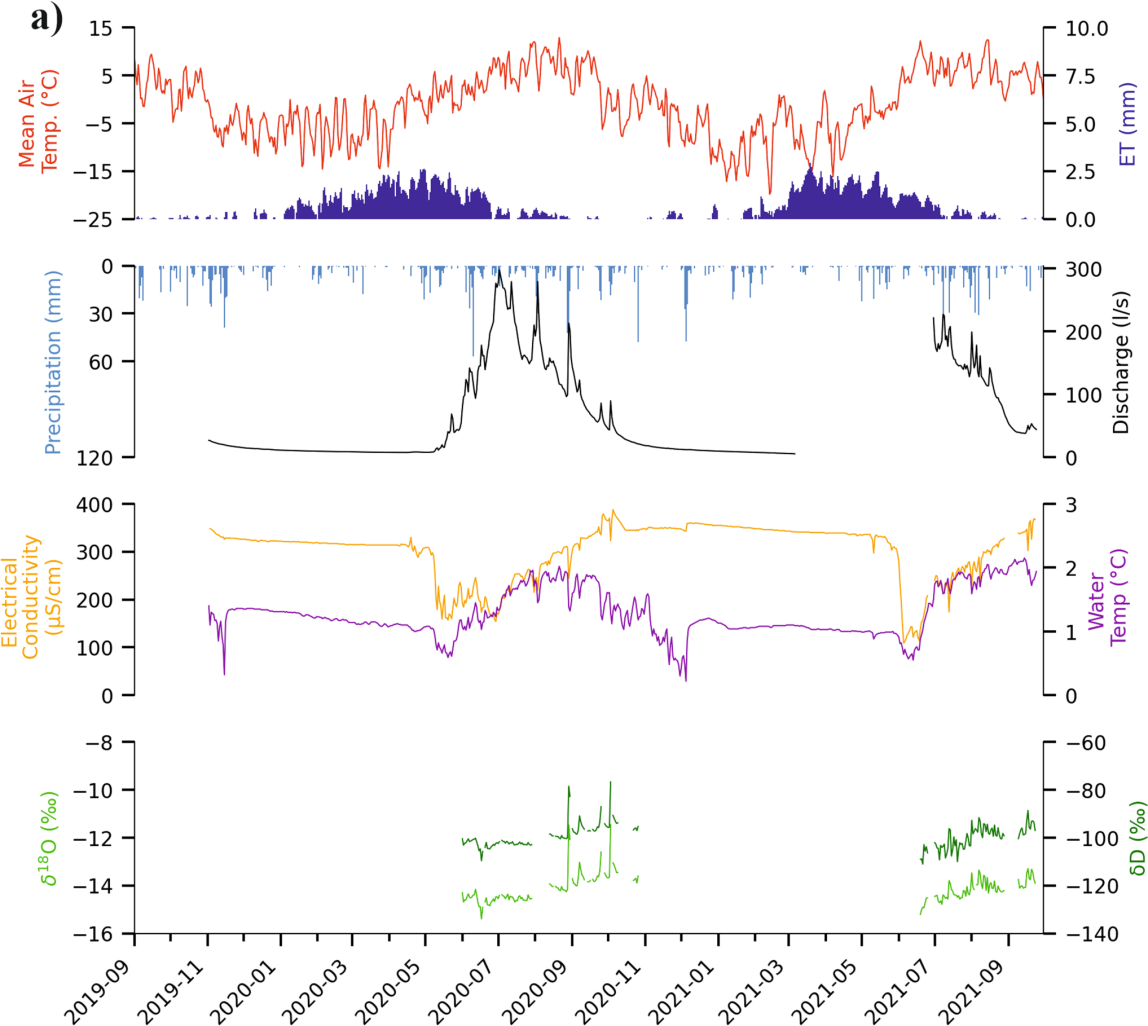

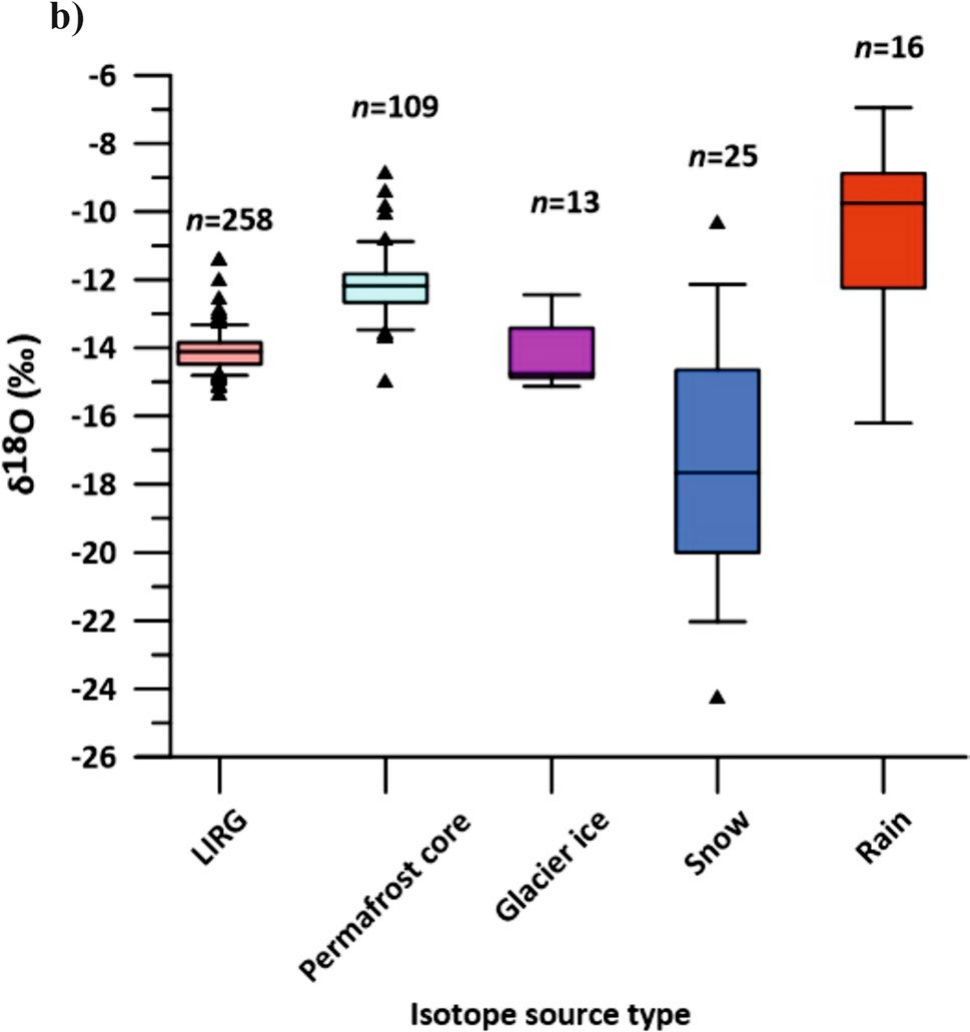
(**A**) Data overview for the period 2019–2021. The complete dataset is presented in Fig. S1 of the ESM. The dataset includes: precipitation and mean air temperature data extracted for the study area from the SPARTACUS dataset (Hiebl & Frei 2016; 2018); estimated potential evapotranspiration (ET0); water temperature, electrical conductivity (EC) and discharge recorded by the data logger installed at the gauging station; isotope data from daily collected samples from LIRG. (**B**) Boxplots representing the isotope signature of LIRG water compared to those of rain, glacier ice and snow samples collected in 2020 and 2021 at locations shown and described in Fig. 1, and the isotope signature of the ice within the permafrost core described by Krainer et al. (2015b). The whisker splits are determined by the 5th and 95th percentiles, with the interquartile range (IQR) and median representing the central portion of the data, while outliers are identified as* triangles* above the 95th percentile or below the 5th percentile; *n* = number of samples

Since 2011 discharge of the active Rock Glacier Lazaun has been measured at a gauging station situated approximately 100 m below the Rock Glacier Lazaun spring (Figs. 1 and 2; Krainer et al. 2015a). Discharge is characterized by distinct seasonal and diurnal variations (Fig. 3; see also Fig. 5.48 and 5.49 in Bertolotti 2022).

### Climate

The mean annual temperature within the Lazaun cirque is around – 2 °C (average mean air temperature obtained from daily minimum and maximum temperature values between 1961 and 2022; SPARTACUS—gridded Spatiotemporal Reanalysis Dataset for Climate in Austria; Hiebl and Frei 2016, 2018), favored by the high altitude and north-northeast orientation of the cirque.

## Materials and methods

Runoff of a spring catchment is directly related to both the recharge with its temporally dominant components (rain, snow and ice melt in (high-) alpine terrain) and the size of the catchment. The delineation of a spring catchment in such settings typically requires that the measured specific discharge align closely with the climatic water balance (CWB), computed as the difference between precipitation and potential evapotranspiration (Haslinger et al. 2023). To define a plausible catchment area for the rock glacier spring in such a hydrogeologically complex environment, a multi-step approach was adopted.

- In the first step, the smallest and the potentially largest catchments and two in between were delineated based on topographic characteristics (*Delineation of potential catchments*).
- For these catchments, the specific discharge was calculated and compared with CWB, including potential glacial and permafrost ice melt water equivalents (*Water balance*).
- A lumped parameter rainfall-runoff model was applied to simulate the runoff dynamics and investigate distinct recharge sources. The model was combined with a semi-distributed snow module and a glacier ice module. The plausibility of the modeling results (recharge components and delineation) was validated using stable isotopes (δ^18^O and δD) and EC to identify the distinct recharge sources, thereby enhancing the reliability and accuracy of the catchment delineation (*Rainfall-runoff modeling*).

### Monitoring and sampling

LIRG has been monitored since October 2011 at the gauging station shown in Figs. 1 and 2, unfortunately with data gaps of months to years due to challenges related to alpine terrain and associated weather conditions. Data loggers were installed for recording water level, water temperature and electrical conductivity (EC) and set to an hourly measurement interval. This study accounts for measurements until September 2021, in alignment with the availability of meteorological data. Because of the distance (approximately 100 m) between the gauging station and the rock glacier spring, water temperature was not analyzed in this study, but measurements from the gauging station are reported in Fig. 3. Regular field campaigns have been conducted from late spring until early autumn and direct discharge measurements have been collected using the salt dilution method (Merz and Doppmann 2006). These measurements allowed to establish rating curves for the quantification of discharge. The rating curve presented by Bertolotti (2022) has been used for the data presented in this study.

Daily precipitation sums and average air temperature data were obtained from the SPARTACUS dataset (Hiebl and Frei 2016, 2018) and averaged for each of the considered catchment areas. Cross-validation results from Hiebl and Frei (2016) indicate that typical relative errors in the SPARTACUS precipitation data are 10–15% at monthly scales, with errors increasing at high elevations and during heavy precipitation events. These uncertainties, evaluated over the 1961–2014 period, primarily affect precipitation estimates while temperature data show considerably lower errors. A few measurements of cumulative rainfall were collected in 2021 at the same rain gauge used to obtain rainwater samples (Fig. 2A; Table S2 of the ESM) and are compared here to the SPARTACUS data. The normalized difference snow index (NDSI), derived from multispectral satellite data, was obtained from the moderate resolution imaging spectroradiometer (MODIS) at 0.5 × 0.5 km spatial resolution to provide an independent check on the simulated snow cover dynamics (v6.1; Salomonson and Appel 2004; Hall and Riggs 2021). Potential evapotranspiration (ET0) was computed using the formula proposed by Oudin et al. (2005), implemented in the PyEt package (Vremec et al. 2024). No direct measurements are available for the actual catchment using on-site measurement devices, such as automatic weather monitoring stations.

Water samples from LIRG were retrieved daily by an automatic water sampler, stored in containers that preserved their isotopic integrity after collection and collected from the sampler during several field campaigns between June and October 2020, and from June to December 2021 (total: 218 samples). These samples have been analyzed for δ^18^O, δD and electrical conductivity. In addition, 25 samples of the snow cover and 16 samples of rainfall water were collected next to the gauging station, glacier ice (melt) samples (13) were collected at the tongue of both cirque glaciers (Fig. 1) and five samples were collected between September and December 2021 also from LGL (Fig. 1) to constrain the isotopic composition of potential recharge endmembers. The glacier ice melt samples were collected at the end of summer, when the glaciers were completely snow-free. Isotope data from the permafrost ice within the rock glacier core Lazaun I (Krainer et al. 2015b) were also considered. Isotopic compositions were reported with respect to the Vienna Standard Mean Ocean Water (VSMOW) (Coplen 1994). The 2020-samples were analyzed for δ^18^O and δD isotopes with the Picarro L2140-i CRDS Isotope and Gas Concentration Analyzer. The 2021-samples were analyzed for the same stable isotopes using continuous flow isotope ratio mass spectrometry (CF-IRMS). Hydrological and stable isotope data are available from Bertolotti and Krainer (2025). An overview of the collected data is presented in Fig. 3. The isotope and EC values of reference samples from each of the assumed recharge components are compared to the isotopes and EC values from the rock glacier spring water, to qualitatively validate the results of the rainfall-runoff model. A local meteoric water line (LMWL) was also calculated (Fig. S3 of the ESM).

To detect periodic daily variations in EC that are not related to snowmelt, periods where the rock glacier catchment is snow-free were inferred using MODIS, SENTINEL, and PLANET satellite data. A spectral analysis of the EC time series within the identified snow-free periods was conducted. This aimed to evaluate the impact of daily fluctuations on the overall EC record during this period, focusing on short-term variation using the Lomb–Scargle periodogram as outlined by Birk et al. (2004). Daily fluctuations in the EC period are an additional indicator of the glacier ice melt as one of the recharge components of the rock glacier spring.

### Delineation of potential catchments

**Fig. 4:**
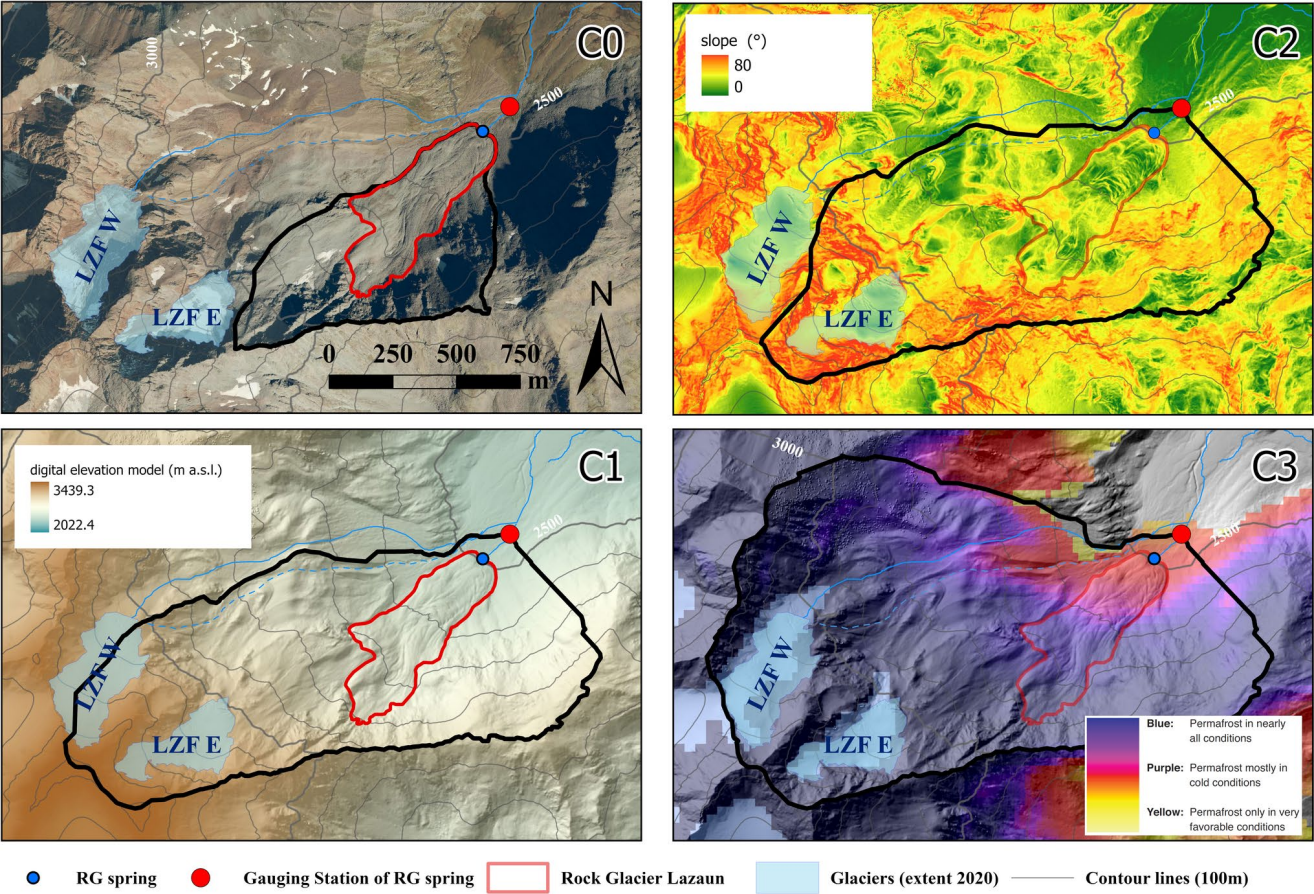
Feasible catchments of the Rock Glacier Lazaun spring. The name of the catchment is written in the upper right corner of each sub-figure, and the catchments are delimited by black boundaries. The C0 catchment also shows the 2020 orthophoto (resolution: 20 cm) of the Autonomous Province of Bozen/Bolzano—South Tyrol (Geokatalog 2021). C1 is underlain by the 2006 high-resolution 2.5 × 2.5-m digital elevation model hillshade provided by the Autonomous Province of Bolzano/Bozen (GeoKatalog 2021). The C2 catchment shows the slope inclination around the Lazaun cirque. C3 is underlain by the Alpine Permafrost Index Map (APIM; Boeckli et al. 2012)

The high-resolution 2.5 × 2.5-m digital elevation model from 2006, provided by the Autonomous Province of Bolzano/Bozen (GeoKatalog 2021), was used as the basis for the topographically based delineation. The delineations and their respective features are as follows (Fig. 4, Table 1):

- C0 is the smallest catchment, which includes the topographic area directly upstream of the rock glacier spring, considering the whole rock glacier as the catchment outlet. These catchment boundaries were considered as the rock glacier spring catchment by Fey and Krainer (2020).
- C1 was delineated purely based on the topography using the hydrological tools of the spatial analyst-hydrology toolbox (“fill”, “flow directions”, “flow accumulation and “watershed” tools) in ArcMap (version 10.6.1) with the gauging station as pour point. Since this resulted in a division of the LZF W, two additional catchment possibilities (C2 and C3) were considered.
- C2 covers a slightly smaller area than C1, excluding the LZF W glacier and the areas strictly related to it. This way, the importance of LZF W for the watershed of the Lazaun rock glacier can be verified.
- C3 considers the whole Lazaun cirque upstream of the gauging station. It includes both cirque glaciers LZF E and LZF W and all glacial and periglacial sediments of possible hydrological relevance in the cirque. This area, however, is also known to be drained by LGL, both by its intermittent and its perennial arms.

**Table 1:** Catchment area, glacier-covered fraction, and rock glacier-covered fraction of each delineated catchment, as shown in Fig. 4

Catchment	C0	C1	C2	C3
Catchment area (km^2^)	0.52	1.42	1.32	2.04
Glacier-covered fraction (%)	0	9.9	5.6	9.0
Rock-glacier-ice-covered fraction (%)	19.3	7.0	7.6	4.9

C1 and C2 also include at least a segment of the intermittent arm of LGL in their boundaries. This was considered both in the CWB calculation and in the model (cf. Rainfall-runoff modeling, parameter *X*_2_).

### Water balance

The specific discharge values (SDM, “specific discharge-measured”) based on data from the gauging station were derived for the hydrological year 2019/20 and all four catchment delineations, where an almost continuous spring flow record was available for comparison (Fig. 3A and Fig. S1 of the ESM). These estimates are obtained by converting the mean discharge in l/s to discharge in mm/year, over the estimated catchment area. The climatic water balance (CWB) was calculated after Haslinger et al. (2023) as the difference between catchment precipitation (directly obtained from SPARTACUS) and potential evapotranspiration (obtained by applying the Oudin formula to SPARTACUS temperature data through the PyEt package by Vremec et al. 2024).

For catchments containing glaciers, a plausible range of glacier ice melt was estimated and added to the catchment water balance (CWB), allowing indirect consideration of the glacier streamflow contribution (stream LGL) within the catchment boundaries. The glacier ice melt estimation was adopted by using the mean annual mass balance of 14 Austrian glaciers and 13 Italian glaciers between 2010 and 2021 (World Glacier Monitoring Service, WGMS 2013, 2015, 2017, 2020, 2021). Minimum, maximum, and mean mass balance resulting from this comparison are also reported in the ESM. For the mass balance derived from the WGMS data no uncertainty is given in the WGMS Bulletins. Therefore, an uncertainty of 10% of the total melt is assumed here, as calculated by Machguth et al. (2008). To obtain the glacier melt contribution per catchment, the mass balance values were normalized by the glacierized area within each catchment, based on the glacial share reported in Table 1 (“glacier-covered fraction”). In addition, a plausible range of glacier ice melt was also roughly estimated based on the annual geodetic mass balance of glaciers LZF W and LZF E. This was done based on the 2005 surface boundaries of the two glaciers (GeoKatalog 2021) and using the difference in elevation in two available digital elevation models (DoD, “DEM of Difference”) of the years 2006 and 2016. The ESM provides the calculated mean geodetic mass balance for both cirque glaciers is reported in Table S1 of the ESM, as well as a corresponding uncertainty analysis.

The rock glacier permafrost ice melting rate per year was estimated following the values given by Krainer et al. (2015b). They state that GPS measurements collected between 2006 and 2012 showed an average melting rate of about 100 mm/year for the frozen permafrost body of the Rock Glacier Lazaun. No DoD analysis was possible for the rock glacier, as the DEMs for 2016 were only available for the actual glaciers and not the entire Lazaun cirque (Geokatalog 2021).

### Rainfall-runoff modeling

A lumped-parameter rainfall-runoff model was employed to (i) identify the dominant recharge components, with a particular focus on the impact of glacier ice melt and (ii) delineate the most plausible catchment area of the rock glacier spring.

**Fig. 5:**
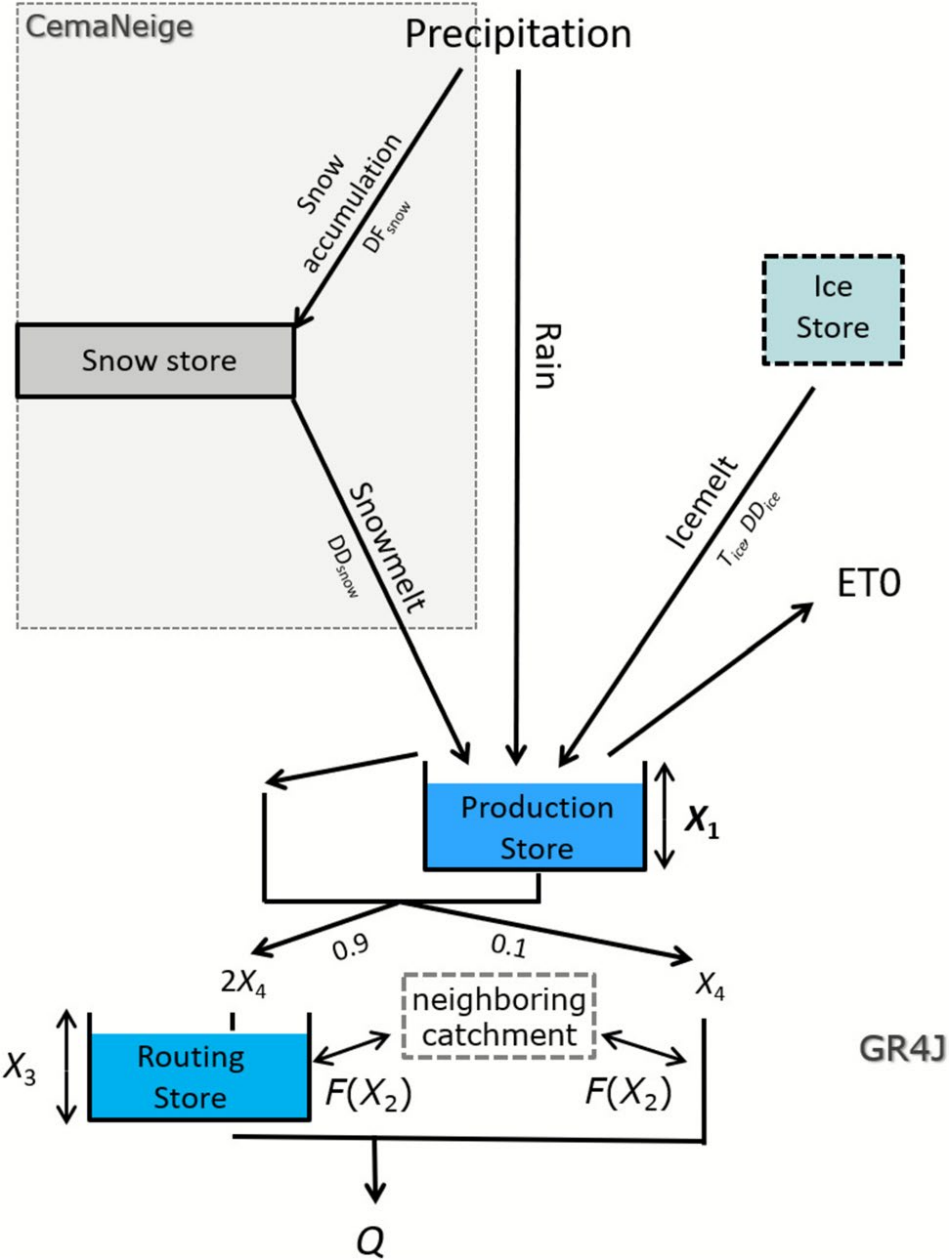
Model setup (modified after Wagner et al. 2021b). *X*_1_ = maximum capacity of the production store; *X*_2_ = water exchange coefficient; *X*_3_ = maximum capacity of the routing store; *X*_4_ = time parameter; *F* = groundwater exchange term acting on the fast and slow flow components; *Q* = runoff simulated by the rainfall-runoff model; DF_snow_ = snow dynamic factor; DD_snow_ = degree-day melt coefficient for snow; *T*_ice_ = temperature at which ice starts to melt (if snow is absent); DD_ice_ = degree-day melt coefficient for glacial ice; ET0 = potential evapotranspiration. The* grey area* emphasizes that the model output is strongly dependent on the catchment area and elevation and the input was normalized over each considered catchment (C1, C2, C3; Fig. 4). Ice melt and ice store refer to the glacier ice melt from LZF E and W

In order to gain insight into the nature of the runoff dynamics, the parsimonious rainfall-runoff model GR4J (Perrin et al. 2003; Fig. 5) was applied to each potential catchment. The model is based on schematized representations of the governing hydrological processes that are represented at the catchment scale. It operates on daily time steps and has proven especially well suited for alpine regions, including the modeling of high-alpine rock glacier springs (Wagner et al. 2016, 2021b). The model input data included daily precipitation, daily average, maximum, and minimum air temperatures, and potential evapotranspiration (Oudin et al. 2005). It consists of two reservoirs: the production storage simulating the soil moisture dynamics, and the routing storage accounting for the groundwater dynamics. They are linked as indicated in Fig. 5. Water is routed between the reservoirs using a unit hydrograph approach, splitting the total flux into 90% delayed and 10% rapid runoff (Perrin et al. 2003). The capacity (mm) of the production storage (*X*_1_) and the routing storage (*X*_3_) depend on the catchment properties. *X*_1_, *X*_3_ and the unit hydrograph time base *X*_4_ are parameters inferred through model calibration. The parameter *X*_2_, referred to as the “groundwater exchange coefficient”, represents net subsurface exchange: positive values indicate water inflow into the catchment, while negative values indicate outflow. In this study, *X*_2_ is used to quantify the fraction of subsurface flow bypassing the spring, with only a small portion likely seeping toward the glacier stream (LGL). A detailed model description is provided by Perrin et al. (2003).

This model was combined with the semi-distributed degree-day snow model CemaNeige that has been designed to simulate snow accumulation and melting processes and tested on a large number of alpine catchments (Valéry et al. 2014; Riboust et al. 2019). Within each potential catchment, snow cover dynamics are simulated independently for five elevation bands of equal area, thereby taking the strong elevation dependence of snowfall and snowmelt into account (Valéry et al. 2014). The model distinguishes between rainfall and snowfall in each of the five elevation bands. It calculates the snowpack’s cold-content and snowmelt using a degree-day method. Two parameters are optimized during the calibration process, to reflect the properties of the studied catchment: the snow dynamic factor DF_snow_ (-), a weighting coefficient for the snowpack's thermal state; and the degree-day snowmelt coefficient DD_snow_ (mm/°C/day), a coefficient governing the rate of snowmelt in response to air temperature. A detailed model description is provided by Valéry et al. 2014.

To adequately represent glacier ice melt in the catchments containing cirque glaciers, the GR4J-CemaNeige model was coupled with an ice-store module, following the approach of Nepal et al. (2017) that was already successfully implemented in the model by Wagner et al. (2021b). The ice-store module is a simple degree-day model simulating the average daily meltwater contribution of the total glacierized area. This ice-store module adds water directly to the GR4J's production store in the absence of snow in the catchment. Its parameters are the temperature threshold *T*_ice_ (°C) for glacier ice melt initiation (if snow is absent), and the degree-day ice melt coefficient DD_ice_ (mm/°C/day), which determines the volume of glacier ice melt per degree of temperature increase. The glacier-covered areal share was kept constant, as the modeling period is relatively short (cf. Wagner et al. 2021b). No attempt is made here to account for permafrost ice melt outside of the rock glacier (see Sect. 4.1).

The model's calibration followed a split-sample test approach, dividing the data into a calibration period (2019–2021) and a validation period (2012–2014), and was performed using the DiffeRential Evolution Adaptive Metropolis (DREAM) Markov chain Monte Carlo (MCMC) scheme (Vrugt 2016). It involved adjusting a total of eight parameters, four parameters of the GR4J model, two of the CemaNeige snow model, and two of the glacier ice-store module. The parameter values obtained through a Bayesian estimation routine to represent effective values describing average catchment characteristics that can be used to assess their plausibility.

## Results

### Water balance

The discharge pattern of Rock Glacier Lazaun is typical for active rock glaciers, as already observed by Krainer et al. (2011). As can be seen in Fig. 3, maximum discharge values reach approx. 300 l/s and are recorded at the beginning of the summer, i.e., when snowmelt contribution is highest. Thereafter, a gradual decrease is recorded from mid-July until October. This corresponds to the slowly diminishing (snow) melt water contribution to discharge. Discharge decrease is interrupted only by isolated peaks, caused by recharge from rainfall events. During winter, discharge is lowest, but not zero. This is also shown by the EC curve, showing constant values of 300–350 µS/cm during winter. Strong daily cycles are recorded during the snow melting season in all three parameters: discharge, water temperature and EC. Water temperature and EC show daily peaks during snow-free periods as well, whereas daily fluctuations in discharge are not so evident (Fig. S2 of the ESM).

Table 2 summarizes the results of the water balance estimates. A plausible estimate of the catchment area was obtained by computing the SDM for each of the four potential catchment delineations depicted in Fig. 4, based on the measured mean annual discharge, and comparing it to their respective CWB. Considering a mean discharge of 55.4 l/s in the hydrological year 2019/2020, the SDM varies from 858 mm/year (largest catchment) to 3380 mm/year (smallest catchment). The SDM of the smallest catchment (C0) exceeds the CWB (885 mm/year) by nearly four times. The SDM of C1 and C2 exceed their respective CWB by approximately 40%. In contrast, the SDM of the catchment C3 is lower than the CWB due to the catchment being too large. However, it shows the best result.

**Table 2:** Specific discharge measured (SDM) for each potential catchment area during the hydrological year 2019/2020 (November 1, 2019, to October 31, 2020), compared to the calculated climatic water balance (CWB) and estimates of glacier and rock glacier permafrost ice melt (in mm w.e.)

Catchment	Statistic	C0	C1	C2	C3
Specific discharge (SDM) (mm/year)		3380	1234	1324	858
CWB (mm/year)		885	895	892	902
Specific glacier ice melt based on WGMS data (mm/year); reported as mean (min and max)	Mean	0	98	55	88
	Min-Max		42–151	24–85	38–137
Specific glacier ice melt based on geodetic mass balance [mm/year] (max loss including uncertainty)	Mean	0	46	24	43
	Max		95	55	93
Specific rock glacier permafrost ice melt (mm/year) based on measurements by Krainer et al. (2015b)	Mean	19	7	8	5
Theoretical total discharge	Mean	912	1000	955	995
	Min-Max		944–1053	924–985	945–1044

Theoretical total discharge = CWB + ice melt (WGMS) + rock glacier permafrost ice melt. This is reported here for a direct comparison to SDM, if glacier ice melt and rock glacier permafrost ice melt are added to the CWB

The calculated values of annual water equivalent of glacier ice melt ranged from – 425 mm/year to – 1516 mm/year, scattering around a mean of – 983 mm/year. These values were converted using the glacial share of the catchments (Table 1) and yield a mean specific ice melt of 98, 55, and 88 mm/year for C1, C2, and C3, respectively. The results and ranges for the individual delineations are listed in Table 2. For comparison, the mean water equivalent of the geodetic glacier mass balance obtained from the DoD was quantified with a loss of – 525 mm/year between 2006 and 2016 for LZF W, and of – 431 mm/year for LZF E. The ESM shows that the geodetic mass balance accuracy was estimated to be about 559 mm. Considering the areal share (Table 1) the maximum values (including the accuracy error) are in the same order of magnitude as the mass balance means based on the values reported by the WGMS (Table 2). Because of the high uncertainty range up to 100%, the obtained geodetic mass balance values were not further considered quantitatively.

Based on the 100 mm/year melting rate of the permafrost rock glacier ice (Krainer et al. 2015b), the water equivalent accounts for less than 1% of the total annual discharge. Since this is considered to lie within the data uncertainty, rock glacier permafrost ice melt was not further considered quantitatively.

### Rainfall-runoff modeling

**Fig. 6:**
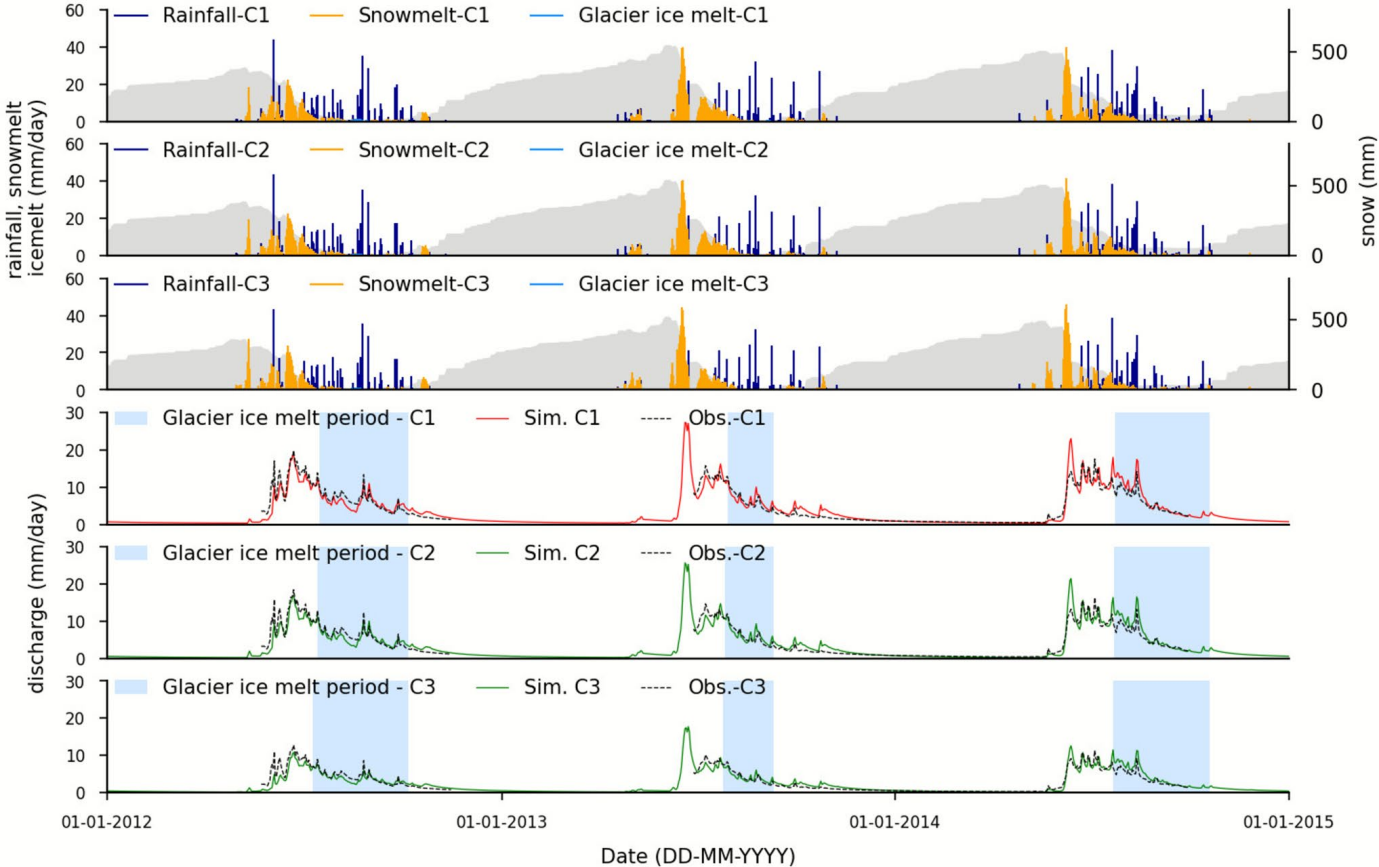
Rainfall, snowmelt, glacier ice melt, and snow accumulation (*in gray*) calculated for the C1, C2, and C3 catchments. In addition, the observed and modeled discharge are depicted. The zoom-in insets show the role of glacier ice melt more clearly. The insect periods correspond to the* blue shaded areas* in the lower three graphs. These highlight periods where glacier ice melt is ongoing, based on the snow-free periods inferred using MODIS, SENTINEL, and PLANET satellite data (cf. Monitoring and sampling)

The water balance analysis of the Rock Glacier Lazaun indicates that the topographic catchment area (C0) of the rock glacier is far too small to explain the rock glacier spring discharge, assuming that 100% of the discharge measured at the gauging station is coming from the actual rock glacier spring (blue dot in Figs. 1 and 2). The model was only applied on the three larger catchment areas C1, C2, and C3 (Fig. 6). For all catchments, discharge decreases gradually over the summer, as the role of snowmelt decreases. During summer discharge responses directly on rain events with sharp peaks. The lowest discharge is recorded in the winter (baseflow), when no recharge occurs because of no glacier melt and because precipitation is stored mainly as snow pack.

The modeled discharge is underestimated compared to the observed one for the period 2012–2013 and slightly overestimated during the rest of the validation period for all catchments (C1, C2, and C3) (Fig. 6). Values of the Kling–Gupta efficiency (KGE) and model bias were calculated for the calibration period (2019–2021) as well as for the validation period (2012–2014), indicated by indices ‘cal’ and ‘val’ in Table 3. Comparing these values serves as a first-order estimate of the model’s robustness and predictive capability (Split-Sample Test; Klemeš, 1986). Note that both KGE and bias indicate strong model performance during calibration and validation for all three catchment delineations (KGE close to 1 and bias close to 0 mm/day). However, similar performance does not guarantee physically plausible parameter sets, as compensation through parameters such as *X*_2_ or added glacier melt may occur. Therefore, the physical plausibility of the model parameters was critically assessed.

**Table 3:** Performance metrics of the modeled discharge series for the calibration period (2019-2021) and the validation period (2012-2014)

Model	C1	C2	C3
KGE (cal)	0.96	0.96	0.95
KGE (val)	0.92	0.92	0.92
Bias (cal, mm/day)	0.0168	0.0009	– 0.0148
Bias (val, mm/day)	– 0.1347	– 0.1581	– 0.0345

The metrics are presented alongside modeled long-term (2011–2021) annual values of glacier ice melt (mm w.e.), snowmelt (mm w.e.), and rainfall for each model. (KGE = Kling–Gupta efficiency, cal = calibration period, val = validation period)

Table 4 summarizes the optimized parameter values for each model run/catchment delineation. The results are compared to typical degree-day values for snow and ice reported in the scientific literature. The production store capacity *X*_1_ varies between 8 and 194 mm, with the model run for C1 showing the lowest values (as expected for a catchment with little to no vegetation). The routing store capacity *X*_3_ ranges from 380 to 494 mm, with the C2 model run showing the lowest value. The unit hydrograph time base *X*_4_ is similar for all model runs (1.2 days). The degree-day melt coefficients for snow (DD_snow_) and ice (DD_ice_) range from 4.6 to 6.6 mm/°C/day. The critical parameter to assess the most likely catchment area appears to be *X*_2_, the groundwater exchange coefficient. This is positive for the C1 and C2 model runs, indicating that both catchments acquire a certain groundwater contribution from nearby catchments. However, it is negative for C3, meaning that this catchment loses a certain amount of groundwater to nearby catchments. This is in accordance with field observations, which show that catchment C3 is not only drained by LIRG but also by LGL (Fig. 1).

**Table 4:** Optimized model parameter values for each model run

Parameter	C1	C2	C3	Literature values
Production store capacity *X*_1_ (mm)	8	15	194	100–1200^a^ 89^b^
Groundwater exchange coefficient *X*_2_ (mm)	7.2	8.5	– 1.6	– 5 to 3^a^ 5.0–9.4^b^
Routing store capacity *X*_3_ (mm)	424	380	494	20–300^a^ 214–303^b^
Unit hydrograph time base *X*_4_ (days)	1.2	1.2	1.2	1.1–2.9^a^ 1.2–1.3^b^
Melt coefficient—snow DD_snow_ (mm/°C/day)	5.1	4.6	6.6	2–12^c^ 2–3^b^
Melt coefficient—ice DD_ice_ (mm/°C/day)	1.1	3.1	1.6	6–20^c^ 7^b^
Snow dynamic factor DF_snow_ (-)	0.90	0.92	0.83	
Threshold temperature *T*_ice_ (°C)	1.0	3.7	2.3	

Literature values refer to (a) surface-water catchments (Perrin et al. 2003), (b) rock-glacier spring catchments (Wagner et al. 2016, 2021b), (c) review of degree-day models by Hock (2003, 2005)

^a^Surface-water catchments (Perrin et al. 2003)

^b^Rock-glacier spring catchments (Wagner et al. 2016, 2021b)

^c^Review of degree-day models by Hock (2003, 2005)

Modeled specific glacier ice melt values (Table 5) can be compared to the specific glacier ice melt values based on data from the WGMS and on geodetic mass balance calculations (Table 2). The model results are relatively close to the WGMS and mass balance data for C2, but are too low for both C1 and C3. While this would indicate that the glacier extent within C1 is too small, the results indicate that within C3 some of the produced glacier melt does not reach LIRG at the gauging station. These results additionally support the results indicated by the negative *X*_2_ values.

**Table 5:** Modeled contribution of glacier ice melt, rainfall, and snowmelt to the recharge of LIRG

Flux	C1	C2	C3
Modeled specific glacier ice melt (mm/year) (%)	22 (2%)	24 (2%)	25 (2%)
Modeled specific snowmelt (mm/year) (%)	599 (56%)	585 (54%)	607 (56%)
Modeled specific rainfall (mm/year) (%)	455 (42%)	465 (43%)	443 (42%)
Modeled total discharge (mm/year)	1076	1074	1072

### Electrical conductivity and stable isotope analysis

**Fig. 7:**
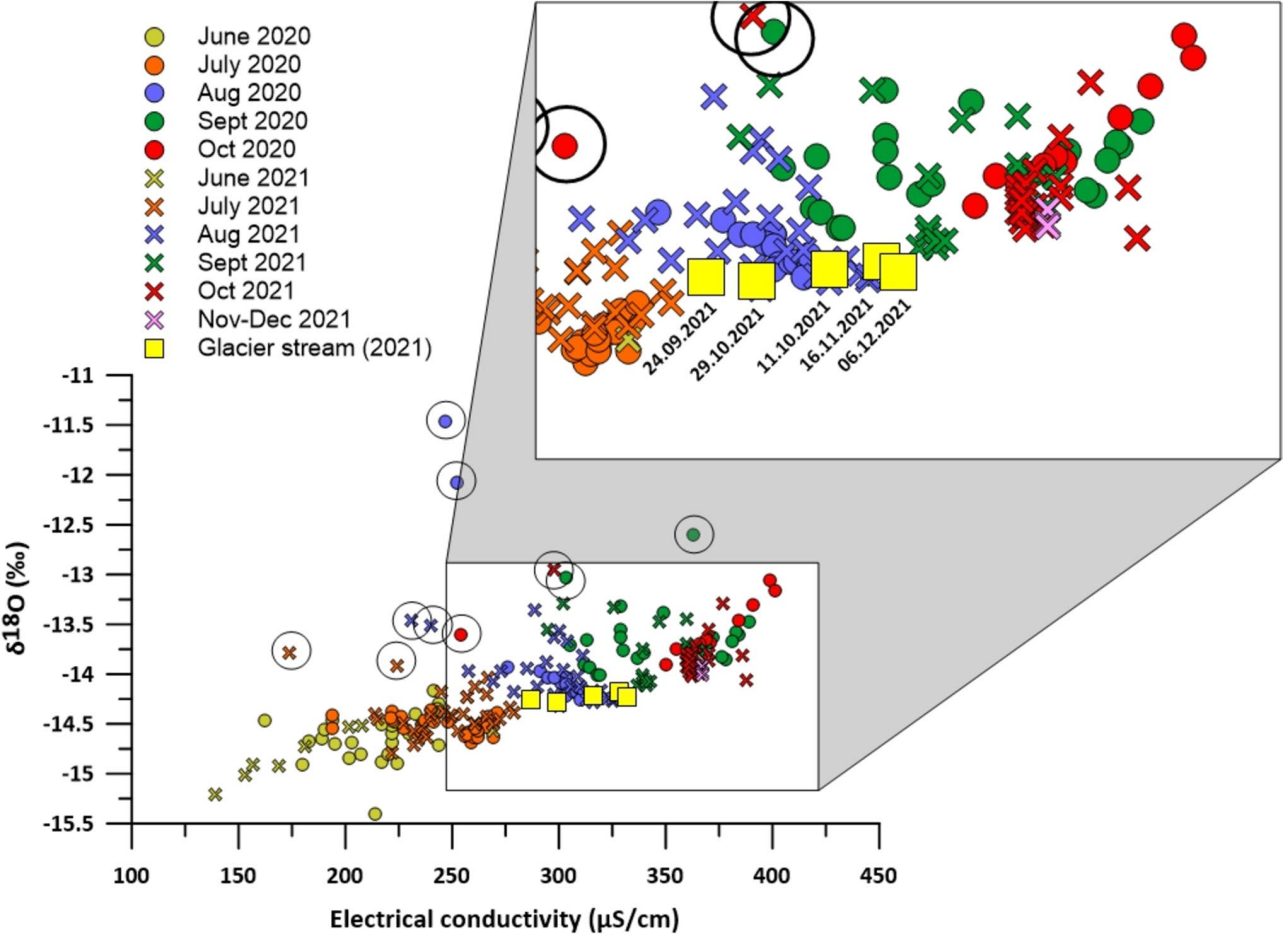
Seasonal oxygen isotope distribution at the rock glacier spring compared to electrical conductivity for 2020 and 2021. The* colors* and related* dates* refer to the sampling date.* Circles* highlight samples affected by rainfall events

Over the summer, electrical conductivity at LIRG increases as the influence of snowmelt – characterized by low electrical conductivity (EC) – declines (Figs. 3A and 7; Fig. S4 of the ESM). In early summer the isotopic signature of snow(melt) is more pronounced. Then isotope values become more enriched in heavier isotopes, reflecting a reduced contribution of snowmelt relative to rainfall. In addition to the rain signal, glacier ice melt may become more prevalent in late summer. The δ^18^O signature of the rock glacier permafrost ice appears scattered over the whole range covered by the samples of LIRG, and cannot be distinctly recognized (“Permafrost core” in Fig. 3B). The δ^18^O and δD signal of LIRG responds markedly to individual rainfall events (peaks in Fig. 3; circled in Fig. 7 and Fig. S4 of the ESM): samples are enriched in heavier isotopes and characterized by lower EC compared to the seasonal trend. In addition, the restricted range of the isotopic signature in rock glacier spring water indicates that it drains a well-mixed system (Fig. 7). While the isotopic signature of the important recharge components rainfall and snowmelt vary considerably (rainfall: – 7.5 to – 15 ‰ δ^18^O; snowmelt: – 10 to – 24 ‰ δ^18^O; Fig. 3B), the isotopic signature of the rock glacier spring water is relatively dampened (– 15 to – 13 ‰ δ^18^O, with peaks up to – 11.5 ‰ δ^18^O; Fig. 3B). This is in line with observations at other active rock glaciers in the Austrian Alps (Heigert 2018; Wagner et al. 2019; Winkler et al. 2021).

The LMWL (Fig. S3 of the ESM) derived from precipitation samples supports the interpretation of the seasonal isotopic evolution at the rock glacier spring (Fig. 7). The LMWL, defined by the relationship δ2H = 7.95·δ1⁸O + 11.89, closely resembles the Global Meteoric Water Line but shows a slightly lower intercept, consistent with local climatic conditions. Rainfall and snowmelt samples show distinct clustering, with snowmelt being significantly more depleted in both δ1⁸O and δD. The rock glacier spring water, by contrast, exhibits a relatively narrow isotopic range along this line, indicating integration and homogenization of diverse water sources within the subsurface system.

Distinguishing the specific contribution of glacier ice melt from the isotope signature alone is more difficult due to the complexity of the system. Recharge components are subject to mixing and storage within the rock glacier and the catchment, and may appear at the spring with variable delays, making it challenging to isolate individual sources. To address the glacier ice melt contribution, a warm and dry week in August 2020 was reanalyzed, when snow was absent from the catchment and permafrost likely buffered from daily energy inputs, although some influence cannot be entirely excluded (data already presented by Bertolotti 2022; Fig. S2 of the ESM). During this period, clear diurnal cycles in EC were observed, with a daily amplitude of approximately 10 µS/cm, suggesting a dilution effect equivalent to 5–10% of total discharge caused by ice melt.

**Fig. 8:**
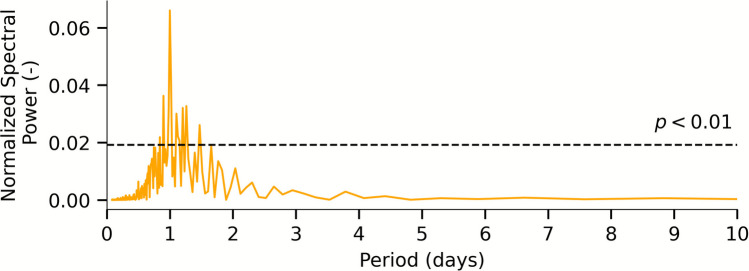
Spectral analysis of the EC hourly time series recorded at the gauging station of LIRG during a snow-free period in late summer 2020 (2020–08-01 to 2020–09–23)

The spectral analysis of EC depicted in Fig. 8 shows the periodogram during the snow-free season in late summer 2020 (01–08–2020 to 23–09–2020). The dominant peak at a period of one day, which is significant at the *ɑ* = 0.01 level, confirms the impact of periodic daily EC variations during this period, suggesting that periodic dilution of high-EC water with less mineralized low-EC water is taking place within the catchment during periods of no snow cover.

## Discussion

### Delineation of the most plausible catchment

The delineation of the recharge catchment is influenced not only by discharge dynamics but also by meteorological input data quality. Before interpreting the results of the rainfall-runoff modeling and catchment delineation, it is crucial to address the key uncertainties and limitations encountered throughout the study. The SPARTACUS precipitation dataset carries uncertainties of 10–15% at monthly scales, with up to 50% errors for daily estimates in complex mountainous terrain (Hiebl and Frei 2018). The cumulative rainfall measurements (Table S2 of the ESM) suggest that the SPARTACUS data for the study area fall well within the uncertainty range. However, precipitation uncertainty in mountain areas—whether from gauging stations or gridded datasets—remains problematic, as the observed atmospheric input signal is often not directly reflected in the hydrological response (Vremec et al. 2025). Beyond the considerable uncertainties in precipitation input, the estimation of ET0 represents an additional source of uncertainty. Empirical approaches—such as the Oudin method, which relies solely on temperature and latitude—are commonly used in rainfall-runoff modeling, but may introduce further uncertainty in catchment water balance estimates, particularly in complex alpine environments where factors such as wind, radiation, and humidity also influence ET0 (Vremec et al. 2024).

Moreover, the subsurface flow paths in the glacial and periglacial sediments are highly complex and largely unknown. This introduces further uncertainty in attributing measured discharge to specific recharge areas, particularly when considering lateral groundwater exchange. Although glacier melt was modeled and geodetic mass balance data were used for validation, no direct measurements exist for LZF W and E, thus introducing more uncertainties. Finally, the simplicity of the GR4J + model, while suitable for data-limited environments, inherently limits its ability to fully represent the spatial variability and complex storage/delay processes in alpine terrain. Low values for parameters like the DD_ice_ may reflect this compensation.

Being aware of these uncertainties, the CWB analysis and model outcomes suggest that:

• the topographic catchment area (C0) of the rock glacier is far too small to explain the rock glacier spring discharge.
• C1 and C2, while showing strong model performance (KGE ~ 0.92–0.96), require substantial groundwater inflow (*X*_2_ > 7 mm) to explain the discharge, suggesting the actual catchment is larger. The model also shows that the glacier extent within C1 is too small compared to the calculated specific ice melts, indicating a larger catchment of LIRG than C1.
• C3, although showing slightly lower modeled glacier melt contributions than expected (Tables 2 and 4), aligns best with the water balance and field observations. The negative *X*_2_ indicates additional groundwater outflow, consistent with the known partial drainage of C3 via LGL. The CWB also suggests a higher plausibility for model run C3, as the SDM is slightly lower than the CWB, suggesting some water within the C3 catchment might be drained elsewhere.

To evaluate the plausibility of the rainfall-runoff model, the model parameter values are compared to typical values reported in the scientific literature (Table 4). Most calibrated parameter values are within reasonable ranges and reflect the physical characteristics of a high alpine catchment. Exceptions are represented by the production store capacity *X*_1_ of C1 and C2, which appear to be too low (8 and 15 mm, respectively) compared to the established range (100–1200 mm). In contrast, the low value of C3 (194 mm) falls within this range and reflects the high alpine environment with poor soil cover and bare rocks. The routing store capacity *X*_3_ is high for all catchments but roughly in line with Wagner et al. (2016, 2021b), considering the widespread sediment accumulation in Lazaun. Thus, the value supports our conceptual understanding of the system. While the DD_snow_ values are plausible, DD_ice_ appears to be too low for all catchments, but reflects the simulated glacier ice melt compared to the calculated glacier ice melt (Tables 2 and 3).

In summary, these results suggest that the most likely rock glacier spring catchment is C3. However, LIRG is not the only stream draining the catchment. A constant groundwater exchange between LIRG and LGL must occur beneath the coarse debris. Glacial meltwater contributes to the annual water balance of the rock glacier spring with about 2% of the total recharge, in line with the water balance for the hydrological year 2019/20 (Table 2).

The model is able to cope with the limited amount of data available in high alpine settings and integrates available knowledge in a parsimonious way. The plausibility checks presented above indicate that the obtained results are roughly in line with independent knowledge of the catchment. However, the conclusions drawn are also limited by the simplicity of the chosen approach and the uncertainty introduced by a complex subsurface geometry and water flow paths, and by the temporal complexity of the dataset (seasonality). Nevertheless, glacier melt water contribution is suggested by the model, the CWB as well as the natural tracer analysis (diurnal EC variations) and the contribution of permafrost ice melt seems to be negligible.

### Recharge components

**Fig. 9:**
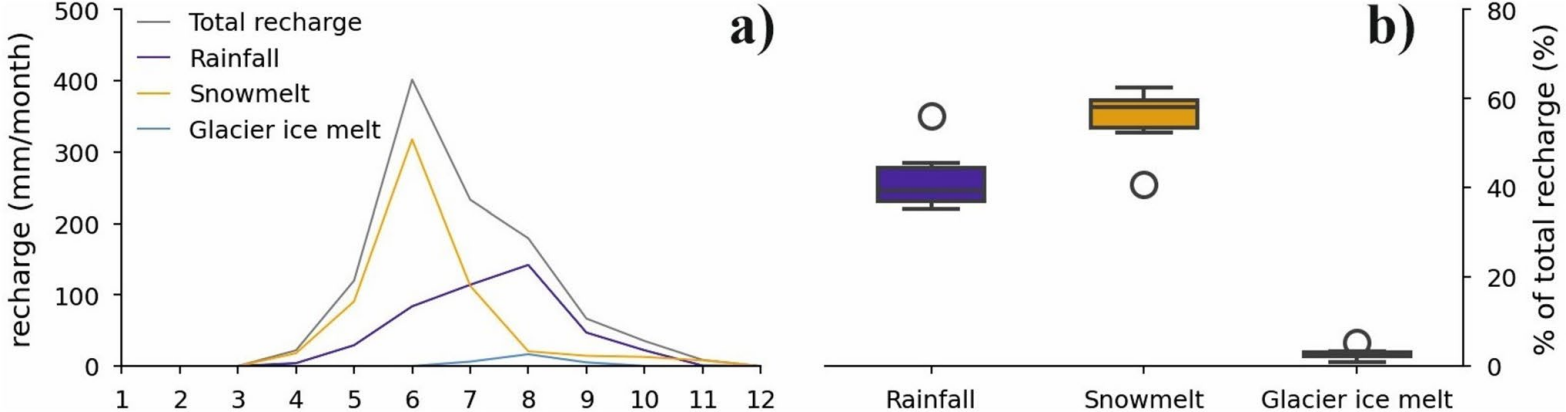
(**A**) Monthly contribution of “recharge” components (input into the rainfall-runoff model); values normalized to catchment area C3. (**B**) The boxplots indicate the annual contributions of individual recharge components to total recharge, expressed as percentages for the years 2012–2021

Figure 9 shows the contribution of the different recharge components for catchment C3. The model shows a stronger contribution of snowmelt (56%) versus rainfall (42%) with a dominant role of snowmelt in spring and early summer and an increasing importance of rain towards the end of the summer. Both the rainfall-runoff models and the water balances also indicate the importance of glacier ice melt: the discharge cannot be explained without glacier ice melt contribution. The glacier ice melt as a recharge component occurs only in the snow-free periods (up to 13%) and accounts for 2% of the total annual discharge. This is in line with the isotopic and EC analysis. These values are distinctly lower than those recorded at Innere Ölgrube (Wagner et al. 2021b), where the amount of ice melt is in the order of 30% of the annual recharge. At Lazaun, the low glacier ice melt contribution obtained from model run C3-Ice (2%) suggests that catchment C3 is too large and the model needs to compensate for this by reducing glacier ice melt (Table 5).

The marked isotopic signal response to individual rainfall events (Figs. 3 and 7; Fig. S4 of the ESM) indicates a rapid system response and the presence of both fast and slow flow components, consistent with previous findings in rock glacier hydrogeology (e.g., Winkler et al. 2018; Jones et al. 2019). In contrast, distinguishing the specific contribution of glacier ice melt from the isotope signature alone (e.g., using a multi-component mixing model) is more difficult due to the complexity of the system. Recharge components are subject to mixing and storage within the rock glacier and the catchment, and may appear at the spring with variable delays, making it challenging to isolate individual sources. The estimated glacier ice melt contribution of 5–10% of total discharge during a warm and dry week in August 2020 (Fig. S2 of the ESM) aligns with the rainfall-runoff model-based assessment, suggesting a similar magnitude during summer months. However, the current natural tracer data set is insufficient for applying a full quantitative mixing model due to the large number of recharge components involved. Another type of tracer is needed to constrain the end members and calculate their contributions. Ongoing data collection will support future efforts to develop a tracer-based mixing model with better-constrained endmembers.

The role of glacier ice melt is confirmed by the daily fluctuation in EC during snowmelt- and rainfall-free period (Fig. S2 of the ESM) and by the spectral analysis result (Fig. 8). The periodic daily EC variations during snow-free periods shown by the spectral analysis can only be reasonably attributed to periodic dilution of the rock glacier spring water by daily glacier ice meltwater cycles (Seelig 2022). This is in line with observations at the Innere Ölgrube rock glacier (Wagner et al. 2021b) where a direct inflow from a glacier stream into the rooting zone of the rock glacier is known. The isotopic signature of both precipitation and rock glacier spring water is very similar to that measured at the Bergli and Ölgrube rock glaciers (North Tyrol, Austrian Alps; Wagner et al. 2021b; Winkler et al. 2021) due to comparable catchment settings. Further, the measured isotopic values of precipitation are in accordance with the regional isotopic signatures of precipitation reported by Giustini et al. (2016) and Longinelli and Selmo (2003, 2010). Field observations support the model result and the natural tracer analysis: it is evident that part of the glacier runoff bypasses the rock glacier and also contributes to LGL.

The general increase in EC towards the end of the summer season along with a decrease in discharge also indicates an increasing groundwater contribution from the well-mixed system. Groundwater is characterized by higher EC and δ^18^O values between – 13 and – 14 ‰, observable during the baseflow period (October–December in Fig. 7) and can be attributed to an unfrozen base layer (Krainer et al. 2015a; Wagner et al. 2021b). The contribution of the groundwater component is also evident when comparing the isotope and EC values of LIRG to those of LGL (yellow squares, Fig. 7). LIRG shows higher EC values and appears to be enriched in heavier oxygen and hydrogen isotopes compared to LGL (only available for autumn 2021; Bertolotti and Krainer 2025). However, LGL isotopic/EC signature indicates a similar but less groundwater affected hydrogeological system (lower EC and more depleted isotopic signature, Fig. 7).

## Conclusions and outlook

This study demonstrates that a simple semi-empirical approach like a rainfall-runoff model, coupled with other modules and supported by natural tracer analysis, helps to delineate the most likely rock glacier catchment. In addition, it allows the quantification of individual recharge components in a complex periglacial and glacial environment. The obtained catchment of the Rock Glacier Lazaun spring is about 2.04 km^2^, encompassing two cirque glaciers that substantially influence the spring flow dynamics. Most of the recharge infiltrates rapidly through the coarse-grained material covering large parts of the catchment. The recharge for the period 2012–2021 was found to be dominated by snowmelt, followed by rainfall and minor glacier ice melt, with average contributions of 607, 443, and 25 mm/year, respectively, corresponding to 56, 42, and 2% of the total recharge. However, exact values are difficult to obtain and should be considered as rough estimates. The contribution of rock glacier permafrost ice melt is within the uncertainty range and therefore not quantifiable. These results are supported by natural tracer analyses including electrical conductivity and stable isotopes. The catchment characteristics deciphered by the rainfall-runoff model are in line with findings of other rock glacier springs in the Eastern Alps.

For a more accurate quantification of recharge-discharge components, it appears necessary to include more precise observations of the other streams within the cirque above the rock glacier. Tracer experiments as well as more detailed hydrochemical analysis (natural tracers) would help to assess the actual impact of both glaciers on the rock glacier spring discharge.

The developed understanding of a complex spring catchment including a rock glacier and cirque glaciers, is important for a better process-based understanding of alpine headwaters. The quantitative identification of the recharge components provides the basis for predicting the impact of climate change on such highly sensitive environments. Ongoing glacier retreat will also change the discharge dynamics of the rock glacier spring.

## Supplementary Information

Below is the link to the electronic supplementary material.

Supplementary file1 (PDF 675 KB)

## Data Availability

The datasets generated during and analyzed during the current study are available in the PANGAEA repository, 10.1594/PANGAEA.979976. The model code and the code used to generate the figures in this study are available upon request from the corresponding author.
