# Supplementary material for: Characterization of recharge components for delineation of a high-alpine rock glacier spring catchment, Ötztal Alps (northern Italy)
Source: Hydrogeol J. 2026 Jan 19;34(3):687–705. doi: 10.1007/s10040-025-03005-y (PMC13083443; doi:10.1007/s10040-025-03005-y)
Supplement: Supplementary file 1 — Supplementary file1 (PDF 675 KB) [file 10040_2025_3005_MOESM1_ESM.pdf]

Hydrogeology Journal: Electronic supplementary material

Characterization of recharge components for delineation of a high-alpine rock glacier spring catchment, Ötztal Alps (Northern Italy)

Giulia Bertolotti[1*,2], Matevž Vremec[2,4], Simon Seelig[2], Karl Krainer[3], Thomas Wagner[2], Andrea Fischer[1], Gerfried Winkler[2]

[1]Institute for Interdisciplinary Mountain Research, Austrian Academy of Sciences, Innrain 25/3, 6020 Innsbruck, Austria

[2]Department of Earth Sciences, NAWI Graz Geocenter, University of Graz, Heinrichstr. 26, 8010 Graz, Austria

[3]Institute of Geology, University of Innsbruck, Innrain 52, 6020 Innsbruck, Austria

[4]Alma Mater Europaea University, Slovenska 17, Maribor, Slovenia

*** Correspondence:**
Giulia Bertolotti
Giulia.Bertolotti@oeaw.ac.at

Calculation of geodetic mass balance accuracy for Lazaunferner East (LZF E) and Lazaunferner West (LZF W)

The accuracy of the calculated geodetic mass balance can mainly be related to the accuracy of the DEMs and the glacier surface area:

DEM2016: 0.5 m horizontal resolution, 0.1 m vertical resolution (more information can be found under: Digitales Oberflächenmodell Gletscher 2016/17 (DOM 0.5 m); metadata in http://geokatalog.buergernetz.bz.it/geokatalog/#!).

DEM2006: 2.5 m resolution, 0.40-0.55 m height accuracy (Knoll & Kerschner, 2009; https://natur-raum.provinz.bz.it/de/digitale-hohenmodelle). No further analysis of the uncertainty could be carried out, as the DEM 2016 only covers glaciated areas. Increasing point densities in modern LiDAR systems usually result in accuracies higher than in 2006 as for example shown for Silvretta (Fischer et al., 2021).

The 2005 glacier surface area accuracy (σ_A) is estimated to be around ± 3.7% according to Knoll & Kerschner 2009, which means an area of 185251 m^2 ± 6854 m^2 for LZF E and 210073 m^2 ± 7773 m^2 for LZF W (GeoKatalog, 2021).

Huss (2013) recommends a density of 850 ±60 kg/m^3 for the conversion of volume to mass balance being appropriate for a wide range of glacier conditions. This value is based on modeling of various conditions including a variable firn cover. As for the glaciers investigated in this study, no firn cover is present anymore. Therefore, the volume change of the DEM 2006 and 2016 within the 2020 borders of LZF E and W was multiplied by a

conversion factor of 900 kg/m^3, mentioned by Huss (2013) as being used for ablation zones. The error derived by this approximation is considered relatively small (Huss, 2013).

The overall estimated uncertainty of the specific geodetic mass balance δb can be calculated as the result of the uncertainty in the conversion factor $\delta\rho$ and the uncertainty of the volume change δv, where the uncertainty of the conversion factor is almost zero. δv is equal to the uncertainty $\sigma_{\delta h}$ derived by the DEMs difference δh (here for example 0.55 m for DEM 2006 and 0.1m for DEM 2016) multiplied by the area A of the glacier, and therefore also dependent on the surface area accuracy σ_A reported above:

$$\delta B = \sqrt{\delta\rho^2 + \delta v^2}$$

Where $\delta b = \delta B/A$ and $\delta v = \sqrt{(\sigma_{\delta h} \times A)^2 + (\sigma_A \times \delta h)^2}$ (After Hugonnet et al., 2021)

For $\delta\rho$ a value of 100 kg/m^3 is assumed (value exaggerated as it is reported by Huss 2013 to be relatively small).

Hereafter we report the detailed calculation:

$$\delta\rho = 100 \text{ kg/m}^3$$

Measured height difference (δh) for the area of LZF W = 583 mm

Measured height difference (δh) for the area of LZF E = 479 mm

$$\sigma_A = 3.7 \text{ \% of } A$$

$$\text{A of LZF E} \pm \sigma_A = 185251 \text{ m}^2 \pm 6854 \text{ m}^2$$

$$A \text{ of LZF W} \pm \sigma_A = 210073 \text{ m}^2 \pm 7773 \text{ m}^2$$

$$\sigma_{\delta h} = \sqrt{0.55^2 + 0.1^2} = 0.559 \text{ m}$$

$$\delta v_{\text{LZF E}} = \sqrt{(0.559 \text{ x } 185251)^2 + (7773 \text{ x } 0.479)^2} = 103622.2213 \text{ m}^3$$

$$\delta v_{\text{LZF W}} = \sqrt{(0.559 \text{ x } 210073)^2 + (6854 \text{ x } 0.583)^2} = 3997607.153 \text{ m}^3$$

$$\delta B_{\text{LZF E}} = \sqrt{100^2 + 103622.2213^{\,2}} = 125897.1561 \text{ m w.e.}$$

$$\delta B_{LZF\,W} = \sqrt{100^2 + 3997607.153^2} = 3997607.155 \text{ m w.e.}$$

$$\delta b_{\text{LZF E}} = 103622.2659 / 185251 = 0.559 \text{ m weq} = 559 \text{ mm w.e.}$$

$$\delta b_{\text{LZF W}} = 125897.1561 / 210073 = 0.559 \text{ m weq} = 559 \text{ mm w.e.}$$

The overall estimated uncertainty of the specific geodetic mass balance δb for both glaciers is therefore 559 mm w.e.

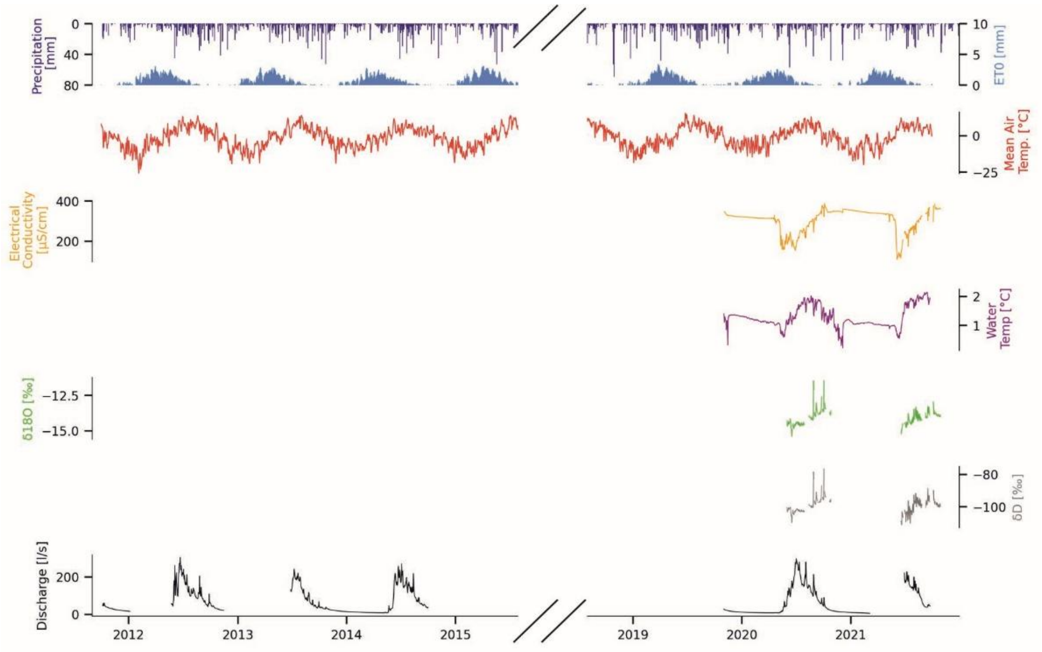

Figure S1. *Dataset overview. Precipitation and mean air temperature data extracted for the study area from the SPARTACUS dataset (Hiebl & Frei, 2016; 2018); estimated potential evapotranspiration (ET0) calculated using the formula proposed by Oudin et al. (2005), implemented in the PyEt package (Vremec et al. 2024); Electrical conductivity and discharge recorded by the data logger installed at the gauging station. Isotope data measured from daily collected samples (See the Monitoring and sampling section).*

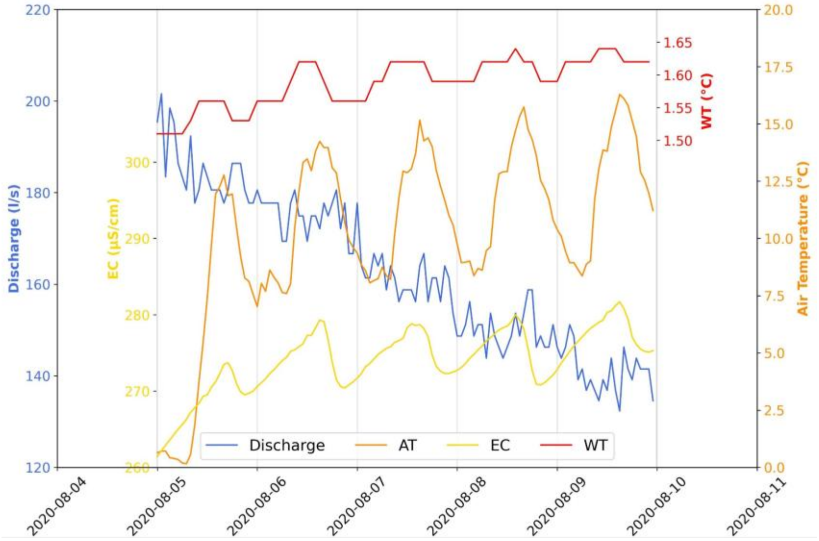

Figure S2. *Hourly resolution of discharge, electrical conductivity (EC), air (AT) and water (WT) temperature for the period August 5th-10th, 2020. EC values are derived from the SEBA logger at the gauging station because of their hourly resolution. For AT, values from the AWS Teufelsegg, corrected with an elevation temperature gradient of 0.69°C/100m for the altitude of the gauging station are displayed. (figure modified from Bertolotti, 2022).*

Table S1. Rock glacier and glacier area information and glacier mass balance. Area values are retrieved from the GeoKatalog (2021) repository. For the geodetic mass balance of both LZF W and E we report the uncertainty only towards a more negative mass balance, since a positive or nearly positive mass balance is unlikely compared to the regional trend and also would not correspond to field observations. These also indicate a visible retreat of both glaciers.

Rock Glacier Lazaun area (m^2)	172465
Lazaunferner East 2020 (m^2)	109304
Lazaunferner West 2020 (m^2)	74511
Lazaunferner East 2017 (m^2)	128911
Lazaunferner West 2017 (m^2)	120843
Lazaunferner East 2005 (m^2)	185251
Lazaunferner West 2005 (m^2)	210073
Lazaunferner East 1997 (m^2)	210439
Lazaunferner West 1997 (m^2)	232299
Mean estimated glacier mass balance (mm w.e./year, 2011-2021) (WGMS)	-983
Min and max of estimated glacier mass balance (mm w.e./year, 2011-2021) (WGMS)	-425 to -1516
Mean geodetic mass balance of LZF W and E (mm w.e./year, 2006-2016)	LZF W: -525 (± 559)<br><br>LZF E: -431 (± 559)

Table S2. Comparison of cumulative rainfall measurements and the rainfall amount from the SPARTACUS dataset for the same time period.

**Time period (yyyy.mm.dd)**	**Cumulative rainfall amount**		**Rainfall amount (SPARTACUS, mm)**
	**measured (ml)**	**averaged over the area of container's opening (5 cm radius) (mm)**	
2021.07.06-08.06	1250	159	161
2021.08.06-28	700	89	76
2021.08.28-09.24	400	51	44

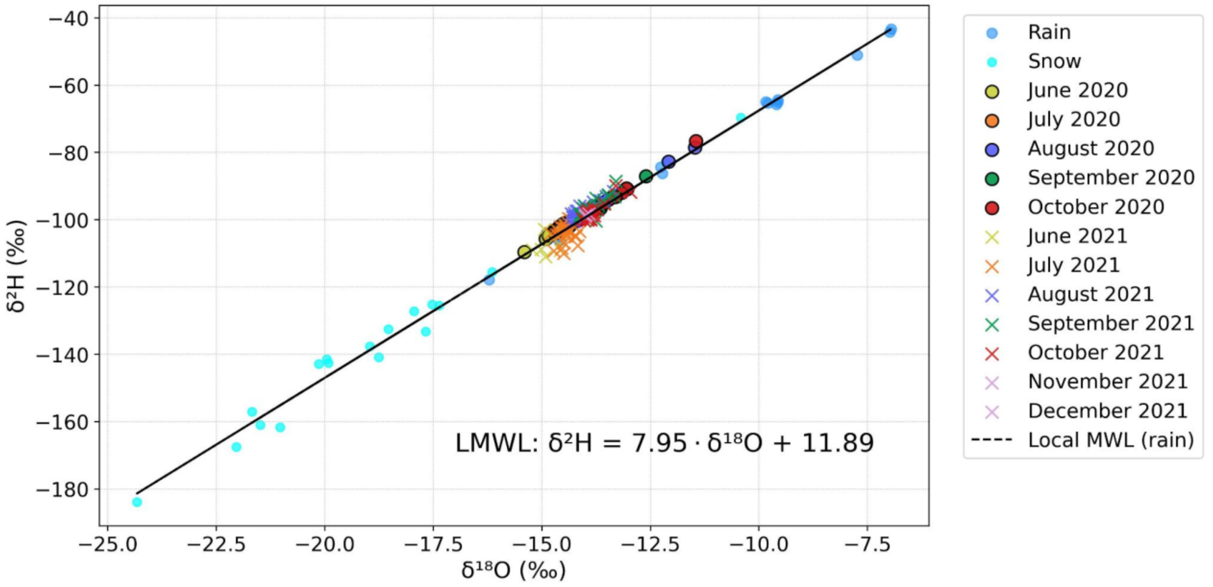

Figure S3. Local meteoric water line (LMWL) calculated based on the analyzed snow and rainwater samples

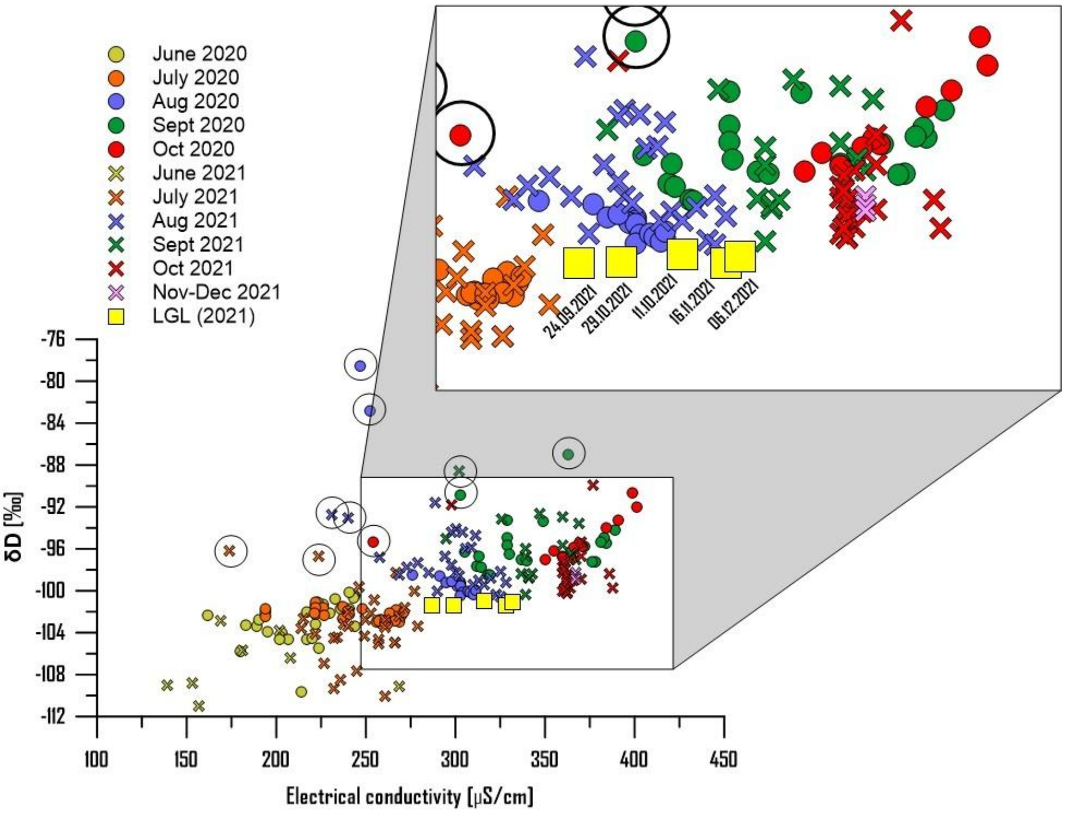

Figure S4. 2020 and 2021 seasonal deuterium isotope distribution at the rock glacier spring compared to electrical conductivity, as a parallel to Fig. 7. The colors and related dates refer to the sampling date. Circles highlight samples affected by rainfall events.
